# Nuclear miR-451a activates KDM7A and leads to cetuximab resistance in head and neck squamous cell carcinoma

**DOI:** 10.1007/s00018-024-05324-x

**Published:** 2024-06-28

**Authors:** Peisong Zhai, Tong Tong, Xiaoning Wang, Chuwen Li, Chun Liu, Xing Qin, Shu Li, Fei Xie, Jiayi Mao, Jianjun Zhang, Haiyan Guo

**Affiliations:** 1 Department of Oral and Maxillofacial-Head & Neck Oncology, Shanghai Ninth People’s Hospital, Shanghai Jiao Tong University School of Medicine, College of Stomatology, Shanghai Jiao Tong University, National Center for Stomatology, National Clinical Research Center for Oral Diseases, Shanghai Key Laboratory of Stomatology, Shanghai, 200011 People’s Republic of China; 2 Department of Oral and Maxillofacial Surgery, Shanghai Stomatological Hospital, Fudan University, Shanghai, 200001 People’s Republic of China; 3 Shanghai Key Laboratory of Craniomaxillofacial Development and Diseases, Fudan University, Shanghai, 200002 People’s Republic of China; 4 Department of Clinical Laboratory, Shanghai Ninth People’s Hospital, Shanghai Jiao Tong University School of Medicine, Shanghai, 200011 People’s Republic of China; 5 Department of Plastic and Reconstructive Surgery, Shanghai Ninth People’s Hospital, Shanghai Jiao Tong University School of Medicine, Shanghai, 200011 People’s Republic of China

**Keywords:** Nuclear miRNAs, Noncoding RNA, Cetuximab, Head and neck squamous cell carcinoma

## Abstract

Cetuximab resistance has been a major challenge for head and neck squamous cell carcinoma (HNSCC) patients receiving targeted therapy. However, the mechanism that causes cetuximab resistance, especially microRNA (miRNA) regulation, remains unclear. Growing evidence suggests that miRNAs may act as “nuclear activating miRNAs” for targeting promoter regions or enhancers related to target genes. This study elucidates a novel mechanism underlying cetuximab resistance in HNSCC involving the nuclear activation of KDM7A transcription via miR-451a. Herein, small RNA sequencing, quantitative real-time polymerase chain reaction (qRT‒PCR) and fluorescence in situ hybridization (FISH) results provided compelling evidence of miR-451a nuclear enrichment in response to cetuximab treatment. Chromatin isolation via RNA purification, microarray analysis, and bioinformatic analysis revealed that miR-451a interacts with an enhancer region in KDM7A, activating its expression and further facilitating cetuximab resistance. It has also been demonstrated that the activation of KDM7A by nuclear miR-451a is induced by cetuximab treatment and is AGO2 dependent. Logistic regression analyses of 87 HNSCC samples indicated the significance of miR-451a and KDM7A in the development of cetuximab resistance. These discoveries support the potential of miR-451a and KDM7A as valuable biomarkers for cetuximab resistance and emphasize the function of nuclear-activating miRNAs.

**Graphical abstract:**

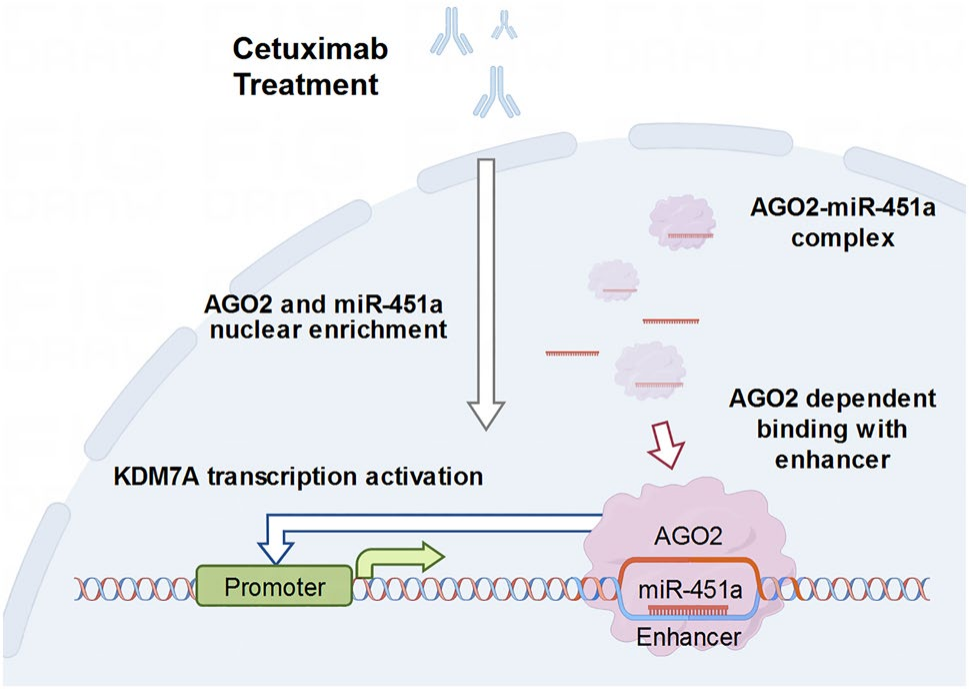

**Supplementary Information:**

The online version contains supplementary material available at 10.1007/s00018-024-05324-x.

## Introduction

Head and neck squamous cell carcinoma (HNSCC) is the main cause of death for malignancies in the head and neck region, with more than 700,000 new cases worldwide [1]. Common treatments for oral squamous cell carcinoma, a common type of HNSCC, include surgery, radiation, and chemotherapy, which yield a survival rate of approximately 50% when applied in combination [2]. Given that 90% of HNSCC patients possess high epithelium growth factor receptor (EGFR) expression, cetuximab, a monoclonal antibody targeting EGFR, was approved for use in HNSCC patients to improve the response rate [3–6]. However, high EGFR expression levels may not guarantee the treatment efficacy of EGFR-targeted therapy [7–9]. Studies have shown that EGFR antibodies used for the treatment of metastatic colon cancer are only 10 to 20% effective [10–12]. The mechanisms of such resistance may be related to EGFR and RAS mutations, mesenchymal–epithelial transition or alternative pathways or downstream pathway activation [8, 13, 14]. However, few efficient strategies corresponding to drug resistance markers and mutations have been identified. A more comprehensive understanding of drug resistance development, in which epigenetic regulation is perhaps a missing key step, is needed.

microRNAs (miRNAs) are involved in the development of many diseases and phenotypes, including cetuximab resistance [15–17]. It is widely believed that miRNAs target the 3'-untranslated region of messenger RNA (mRNA) and negatively regulate gene expression posttranscriptionally in the cytoplasm [18]. However, various other possible mechanisms have been proposed [19–21], among which miRNAs activate gene expression in the nucleus by binding to promoters [22, 23] and enhancers [24–26]; this process is known as RNA activation (RNAa). The miRNAs that engage in this mechanism are named nuclear activating miRNAs. It has been reported that RNAa can modulate disease development, including tumorigenesis [27–29]. Huaping Li et al. elucidated a nuclear activation mechanism involved in cardiomyocyte dysfunction [30]. Overexpressed miR-320 directly targets and activates the promoter of CD36, a fatty acid translocase, which induces cardiac lipotoxicity. In another study, Ying Liang et al. reported that the loss of the enhancer switch miR-339 in breast cancer diminishes the expression of the tumour suppressor gene GPER1, thus facilitating tumorigenesis [25]. These results indicate that the regulatory machinery within the nucleus is not fully understood and may complement miRNA function.

To further investigate the role of miRNAs in cetuximab resistance in HNSCC and whether any nuclear miRNAs facilitate this process, in this study, we identified a mechanism by which nuclear miR-451a activates KDM7A to promote cetuximab resistance. With respect to cetuximab treatment, miR-451a was enriched in the nuclei of cetuximab-resistant HNSCC cells. We utilized chromatin isolation by RNA pulldown and microarray combined with bioinformatics analyses for nuclear miR-451a target mining. The results revealed enhancer-miRNA interaction loci within the tenth intron of the KDM7A gene. This process was further confirmed to be dependent on nuclear AGO2, a miRNA-binding protein that promotes nucleic acid interactions. Through clinical sample analyses, further experiments demonstrated that miR-451a and KDM7A contribute to HNSCC cetuximab resistance. These results reveal a vital and unique role of nuclear activating miRNAs, which is inspiring for new strategies to help overcome cetuximab resistance by targeting either miRNAs or target genes.

## Materials and methods

### Cell culture and cultivation of a cetuximab-tolerant strain

The human tongue cancer cell line CAL27 and human embryonic kidney 293 T (HEK-293 T) cells were purchased from American Type Culture Collection (ATCC), and WSU-HN30 (HN30) cells were kindly provided by the University of Maryland Dental School, USA. The cells were cultured in high-glucose Dulbecco’s modified Eagle’s medium (DMEM) (BasalMedia, China) supplemented with 100 U/mL penicillin and 100 µg/mL streptomycin supplemented with 10% foetal bovine serum in a humidified 37 °C incubator with a 5% CO_2_ atmosphere. To generate cetuximab-tolerant cell strains, we cultured CAL27 and HN30 cells with cetuximab via a stepwise incremental approach. The concentration of cetuximab started at 5 μg/mL and increased sequentially to 20 μg/mL. Then the concentration increased from 25 to 300 μg/mL. The cells were not passaged until they could grow stably under increasing concentrations of cetuximab and yielded the same proliferation rate as the parental cells without cetuximab. Cetuximab-resistant cells eventually stabilized at 300 μg/mL, and the concentration maintained at the culture concentration.

### Isolation of nuclear and cytoplasmic RNA

Separation of the nuclei and cytoplasm was performed using the PARIS™ Kit (Thermo Fisher Scientific, USA) for RNA quantification. A total of 2 × 10^7^ cells were divided in half and treated with disruption buffer to extract total RNA and fraction buffer for proportional RNA. After centrifugation, the fractioned cell lysate was separated into suspended nuclei and the supernatant cytoplasmic portion. The precipitated fraction was then treated with disruption buffer to obtain nuclear lysates. Three samples were then subjected to RNA extraction using the miRNeasy Micro Kit (QIAGEN, Germany). The samples were then reverse transcribed, and qRT‒PCR was performed. MALAT1, NEAT1, TUG1, and U6 were selected as endogenous control genes for the nuclear fraction, and BIRC5 and ACTB were selected as endogenous control genes for the cytoplasmic fraction.

### RNA extraction and quantitative real-time fluorescent polymerase chain reaction (qRT‒PCR)

Total RNA was extracted using TRIzol reagent (TaKaRa, Japan) according to the manufacturer’s protocol. The extracted total RNA was evaluated by using a NanoDrop 2000 (Thermo Fisher Scientific, USA) and reverse transcribed into cDNA using a PrimeScriptTM RT Reagent Kit (TaKaRa, Japan), and qRT‒PCR was performed using Hieff UNICON® qPCR SYBR Green Master Mix (High Rox) reagent (Yeasen, China). For miRNA, reverse transcription was performed using the miRcute Plus miRNA First-Strand cDNA Kit (TIANGEN, China), and qRT‒PCR was performed with the miRcute Enhanced miRNA Fluorescence Quantitative Assay Kit (SYBR Green) (TIANGEN, China). The qRT‒PCR results were analysed using the 2^−ΔΔCt^ method. qRT‒PCR primers were synthesized by Sangon Biotech (Shanghai) Co., Ltd. (China). All primer sequences are listed in the supplementary material (Table S1). The U6 primer for miRNA endo-reference was obtained from TIANGEN Biotech (Beijing) Co., Ltd. (China).

### Small RNA sequencing

RNA extraction and library construction were performed as follows. Small RNA was extracted using a mirVana miRNA Isolation Kit (Ambion, USA) according to the manufacturer’s protocol. RNA quantification was carried out using a Nanodrop 2000 instrument (Thermo Fisher Scientific, Inc., USA). The integrity was determined by an Agilent 2100 Bioanalyzer (Agilent Technology, USA). A total of 5 μg of small RNA per sample was used for small RNA library construction using TruSeq Small RNA Sample Prep Kits (Illumina, USA.). Briefly, small RNAs were ligated to adapters at each end. Then, the adapter-ligated RNA was reverse transcribed to cDNA, and PCR amplification was performed. PCR products ranging from 140–160 bp were isolated and purified as small RNA libraries. Library quality was assessed on an Agilent Bioanalyzer 2100 system using DNA high-sensitivity chips. The small RNA libraries were finally sequenced using the Illumina HiSeq 2500 platform. Reads containing 50 bp (single-end reads) were generated.

### Fluorescence in situ hybridization (FISH) assay

In situ hybridization of miR-451a was performed with both cells and paraffin-embedded tissue sections. FISH for cell samples was conducted using a fluorescent in situ hybridization kit (RiboBio, China). Specifically, samples in confocal dishes were fixed in 4% paraformaldehyde and permeated with 0.5% Triton-100X. After being washed properly with PBS, the specimens underwent prehybridization for 30 min and were then hybridized with a Cy5-labelled miR-451a probe (RiboBio) overnight at 37 °C under humid, dark conditions. The following procedures were performed in the dark. The specimens were washed with washing buffer and PBS several times and restained with DAPI. The samples were imaged using confocal microscopy (Nikon A1, Japan). Embedded sections for FISH were boiled for digestion and treated with 3% hydrogen peroxide for endogenous peroxidase blockade after dewaxing and dehydration. The remaining hybridization and DAPI staining steps were conducted as described above.

### Lentivirus transduction and the CRISPR/Cas9 system

miR-451a overexpression and KDM7A knockdown by shRNA were performed via lentivirus transduction according to the manufacturer’s instructions with 5 μg/ml polybrene. After 12 h of transduction, puromycin (10 μg/ml) was used to select stably transduced strains. The miR-451a overexpression sequence is its mature form (AAACCGTTACCATTACTGAGTT). The shRNA sequences are provided in the supplementary material (Table S2). A CRISPR/Cas9 vector was constructed by Zorin Biotech Co., Ltd., and transduced into CAL27_CTX cells to knock out the miRNA-targeted enhancer region (small guide RNA (sgRNA) sequence 5’-CGTGTGAATGCCAGTAGTAGAGG-3’).

### Chromatin isolation by RNA purification and sequencing (ChIRP-seq)

ChIRP was carried out based on a standard ChIP assay using a SimpleChIP® Enzymatic Chromatin IP Kit (Cell Signaling Technology, USA). Briefly, CAL27_CTX and HEK-293 T cells were treated with 300 μg/mL cetuximab for at least 48 h and then transfected with biotinylated miRNA-451a (RiboBio, China). Within 48 h, the cells were prepared by crosslinking with formaldehyde. Chromatin was segmented using micrococcal nuclease, and cell lysis was carried out via sonication to break the nuclear membrane. Total segmented sample (20 µL) was collected after centrifugation and sample dilution. The remaining segmented chromatin was then incubated with streptavidin magnetic beads (Merck, Germany) overnight at 4 °C. The samples were collected with a magnetic stand and washed several times before elution. The eluted samples were reverse crosslinked and subjected to DNA purification. Finally, each DNA sample was eluted in a total volume of 30 µL twice and stored with templates at − 20 °C before sequencing.

A total of 20 ng of DNA template was used to construct libraries for ChIRP-seq. For quality control of the sequencing data, the Trimmomatic, BLASTN and NT databases were used. Bowtie2 was used to align the reads to the human genome. Strand cross-correlation (SCC) was used to assess the ChIRP pull-down quality. MACS2 was used to call the peaks (*q* value <  = 0.05), with enriched target regions calculated by an algorithm. Gene symbols and other information were annotated to the peaks by chipseeker (v1.12.1). MEME-ChIP was used to analyse the motifs in the peaks, and Tomtom was used to compare the motifs and to annotate them. Finally, Manorm (v 1.18.0) was used to analyse differences in peak reads between groups (*p* value < 0.01 and log2(*M* value) > 1). Statistical significance was analysed by the R package DiffBind. miRNA binding targets among the peaks were predicted by miRanda (total score > 100). Enhancer regions and superenhancers among the peaks were predicted by rank ordering of superenhancers (ROSE), and the overlapping genes, proximal genes and closest genes were annotated. Peaks of statistical significance that were predicted to be miR-451a targets and annotated as enhancers were considered potential target regions of miR-451a.

### Microarray

In this study, four samples were collected for microarray analysis. CAL27 was used as a control for CAL27_CTX. The miR-451a-overexpressing CAL27 cells were compared with the corresponding CAL27 NC cells (the empty vector for miRNA expression). Shanghai OE Biotech Co., Ltd. (China) analysed the four samples using the Agilent SurePrint G3 Human Gene Expression v3 8 × 60 K Microarray platform. Total RNA was quantified using a NanoDrop 2000, and RNA integrity was assessed using an Agilent Bioanalyzer 2100. Standard protocols for sample labelling, microarray hybridization, and washing were followed. The data were analysed using Feature Extraction software (version 10.7.1.1, Agilent Technologies, USA) to obtain the raw data, which were then normalized with the quantile algorithm. Differentially expressed genes were identified based on fold change and *P* value calculations obtained through the *T* test (fold change ≥ 2.0, *P* value ≤ 0.05). GO analysis and KEGG analysis were performed to determine the roles of the differentially expressed mRNAs. Hierarchical clustering was used to visualize gene expression patterns among the samples.

### Western blotting

Total cell proteins were prepared with sodium dodecyl sulfate (SDS) lysis buffer (Beyotime, China). Proteins were separated by 4–20% polyacrylamide gel electrophoresis and transferred onto 0.22-μm PVDF membranes (Merck Millipore, USA). The blots were blocked with 3% skim milk for 1 h at room temperature and then incubated with primary antibodies overnight at 4 °C and with secondary antibodies for 1 h at room temperature. The signal was observed using ECL Ultra (New Cell and Molecular Biotech, Suzhou, China). The antibodies used are listed in supplementary material Table S3.

### Dual luciferase reporter assay

The luciferase reporter gene plasmids pGL3-basic-h-KDM7A-TAR and pGL3-basic-h-KDM7A-INT (vector structure PGK-luc2-MCS-SV40-Rluc) with the predicted miR-451a target region and the intron in which it is located, the control plasmid pGL3-basic-NC and the positive control pGL3-basic-Promoter were synthesized by Zorin Biotech. The Renilla luciferase reporter plasmid pGMR-TK was synthesized by Genomeditech (Shanghai, China). The target plasmid was cotransfected with the Renilla plasmid and miR-451a mimic (or scrambled NC as a control) into HEK-293 T cells. Cell lysates were extracted 48 h after transfection, and relative light unit (RLU) was detected using a Dual Luciferase Reporter Gene Assay Kit (YEASEN, China). The luciferase RLU was first normalized to Renilla luciferase activity and then normalized to data from the control group, which was transfected with the scrambled mimic NC. Each group was prepared in triplicate.

### MTT assay and colony formation

MTT and colony formation assays were performed to characterize cell proliferation. After 6 h of siRNA transfection, the cells were seeded into 96-well plates at a density of 1000 cells per well in triplicate. After the cells adhered and fully unfolded, MTT reagent (Yeasen, China) was diluted ten times and added to each well. The cells were subsequently incubated for 4 h at 37 °C. After carefully removing the supernatant, the formazan produced was dissolved in 150 μL of dimethylsulfoxide (DMSO), and the optical density of the solution was measured at 490 nm using a microplate reader (SpectraMax i3, Molecular Devices, USA). Colony formation assays were performed together with MTT assays. Cells were seeded into 6-well plates at a density of 1000 cells per well and incubated for ten to fourteen days to form cell colonies. The colonies were fixed, stained with crystal violet, and dried. Colonies were scanned using a Canoscan 5600F (Canon, Japan) and counted using ImageJ v1.52.

### Transwell assay and wound healing assay

Transwell assay was performed using 24-well Transwell chambers with 8-μm porosity polycarbonate filters (Corning, USA). 200 μL cell suspension with 1 × 10^5^ cells in serum-free medium was added into each upper chamber, while 500 μL of DMEM supplemented with 20% FBS was added to the lower chambers as a chemoattractant. After incubating for 24–48 h, the cells were fixed with 4% paraformaldehyde (Sangon Biotech, China) for 15 min and stained with 1% crystal violet (Sangon Biotech, China) for 15 min. The cells on the upper surface of the filter were carefully removed. Images were taken using an inverted phase-contrast microscope at 200 × magnification. The cell area was calculated with ImageJ (v.1.52, USA). The wound healing assay was conducted in a 6-well plate with 90% confluence rate of sample cells. A scratch was made on the surface using a pipette tip. The cells were cultured in high-glucose DMEM without FBS until the end of the experiment.

### siRNA and plasmid transfection

Small interfering RNAs (siRNAs) were synthesized by Zorin Biotech Co., Ltd. (China). The siRNA sequences are listed in the supplementary material (Table S2). The siRNAs were transfected with Lipofectamine^TM^ 2000 (Invitrogen, USA) according to the manufacturer’s instructions. Before transfection, the cells were counted and cultured in 6-well plates at 0.25–1 × 10^6^ cells per well. The final concentration of siRNAs per well was 100 pmol/L. The plasmid for overexpressing KDM7A (CMV-hKDM7A-3 × Flag-EF1α-copGFP-T2A-puro) and the empty vector were synthesized by Zorin Biotech Co., Ltd. All plasmids were transfected with Lipofectamine^TM^ 3000 (Invitrogen, USA) according to the manufacturer’s protocol.

### Subcutaneous tumour xenograft formation

Subcutaneous xenografts in BALB/C nude mice were utilized to evaluate the oncogenicity and cetuximab resistance of CAL27 and CAL27_CTX cells under each experimental condition. In brief, 5 × 10^5^ cells were resuspended in 50 μL of serum-free culture medium and mixed well with 50 μL of highly concentrated Matrigel (Corning, USA) on ice. The cell suspensions were quickly injected subcutaneously into both flanks of BALB/C nude mice (six weeks old). After solid tumour formation, the tumour volumes were measured every three days. Cetuximab was administered intraperitoneally (1 mg/mouse). The mice were sacrificed at approximately 30 days, and the transplanted tumours were removed for weight measurement, H&E staining, and IHC analysis. The antiago-miR-451a product was purchased from RiboBio (China) and was used according to the manufacturer’s protocol.

### Bioluminescent imaging

The xenograft tumours for in vivo bioluminescent imaging were pre-stained with DiD (Beyotime, China) at a concentration of 10 μM. After 15 min of staining, the cells were washed once with PBS, and the cell suspension was centrifuged (500 × g, 3 min) again to collect the cells. Bioluminescent imaging in this study was performed using PE IVIS Spectrum (PerkinElmer, USA), where mice were first anaesthetized with isoflurane and subsequently placed into the in vivo imager. Imaging data were analysed using Living Image software.

### Immunohistochemistry (IHC) assay

In brief, excised xenograft tumour samples were fixed in 4% paraformaldehyde and embedded in paraffin prior to being sliced. The slices were dewaxed and rehydrated, and antigens were retrieved and blocked before incubation with primary antibodies and secondary antibodies. After colour development and haematoxylin nuclear counterstaining, the slices were dehydrated and sealed. Images were scanned with a light microscope (Olympus, Japan) at 10 × 40 magnification and quantified using Image-Pro Plus. Five random fields were examined per sample.

### RNA pull-down assay

CAL27 and CAL27_CTX cell precipitates were collected and lysed for nucleic acid extraction according to the SimpleChIP® Enzymatic Chromatin IP Kit (Cell Signaling Technology, USA). Nuclei were lysed, and chromatin was segmented for further experiments. The miRNA probe for miR-451a was the same as that used for ChIRP from RiboBio. The probes were washed and preincubated with streptavidin beads. The nucleic lysates from CAL27 and CAL27_CTX cells and RNA-bound beads were incubated in RNA‒protein binding reaction master mix according to the instructions of the Pierce™ Magnetic RNA‒Protein Pull-Down Kit (Thermo Fisher Scientific, USA) at 4 °C for 60 min. RNA conjugates were then washed and eluted for further Western blot analysis.

### Chromatin immunoprecipitation (ChIP)

ChIP was performed using a SimpleChIP® Enzymatic Chromatin IP Kit (Cell Signaling Technology, USA) according to the manufacturer’s instructions. Rabbit IgG was used as a negative control, an anti-H3K27ac antibody was used to identify enhancers in the genome, and an anti-AGO2 antibody was used to investigate the interaction between AGO2 and chromatin within the nucleus (the antibodies used are listed in Table S3). The DNA fragments were purified and analysed by qRT‒PCR. The results are presented as fold changes. The primers used are listed in Table S1.

### Isolation of nuclear and cytoplasmic proteins

Nuclear and cytoplasmic separation for protein detection was performed using a Nuclear and Cytoplasmic Protein Extraction Kit (Beyotime, China) according to the manufacturer’s instructions. In brief, the cells were scraped and collected by centrifugation. The cytoplasmic and nucleic lysates were fractioned sequentially and treated as described in the previous section for western blot assays.

### Patients and specimens

Tumours from 87 confirmed HNSCC patients with available cetuximab treatment records were collected from the Department of Oral and Maxillofacial-Head and Neck Oncology, Ninth People’s Hospital, Shanghai Jiao Tong University School of Medicine (Shanghai, China). The tumours were stored in RNAprotect Tissue Reagent (Qiagen, Germany). RNA was extracted using TRIzol reagent, followed by qRT‒PCR analysis as previously described.

### Statistical analysis

Statistical analysis was performed using GraphPad Prism 9.0.2 and R 3.6. The data are presented as the mean ± standard deviation (SD). Differences between two groups were analysed by Student’s *t* test. Differences among more than two groups were analysed by one-way analysis of variance (ANOVA) or two-way ANOVA. Differences for which the *P* value < 0.05 were considered statistically significant. Logistic regression analysis of clinicopathological data from HNSCC patients was performed in R. *P* < 0.05 was considered to indicate statistical significance.

## Results

### Establishment of a cetuximab-resistant HNSCC cell line

To better investigate the effect of cetuximab resistance in HNSCC, we compared the survival rates of different HNSCC cell lines and constructed cetuximab-tolerant CAL27 and HN30 cell lines for further analysis. The results showed that CAL27 and HN30 cells were more sensitive to cetuximab than were SCC9 or SCC25 cells (Fig. S1). Therefore, CAL27 and HN30 cells were treated with cetuximab in a stepwise manner to construct a cetuximab-tolerant cell line. As the concentration of cetuximab increased, the survival rates of the cetuximab-resistant CAL27 and HN30 cell lines (hereafter referred to as CAL27_CTX and HN30_CTX, respectively) were increasingly lessened by cetuximab, especially at a concentration of 300 μg/mL, which was determined to be the culture concentration for CAL27_CTX and HN30_CTX in our experiments (Fig. S2a, b, e and f). The cell growth curve of the CAL27 and HN30 cells sensitive to cetuximab treatment was much lower than that of the control cells on days three and five (Fig. S2c, g). Accordingly, CAL27_CTX cells did not exhibit differences in growth rate, colony number, or migration area even after treatment with 300 μg/mL cetuximab (Fig. S2d, h, i-t), indicating that the cetuximab-resistant cell lines were successfully established. We also conducted amplification and exon sequencing of EGFR in CAL27 and HN30 cells and in CAL27_CTX and HN30_CTX cells to investigate EGFR mutations. The results showed that the cetuximab-resistant cells had mutations completely identical to those in their parental cell lines (see supplemental material file amplification exon seq.xlsx), indicating that the acquired resistance to cetuximab did not result from new EGFR mutations.

### miR-451a is significantly enriched in the cetuximab-resistant cell nucleus

To investigate epigenetic regulation, especially the role of miRNAs in cetuximab resistance, we utilized small RNA sequencing for miRNA detection. To better distinguish the subcellular location of these miRNAs, sequencing was combined with cell nucleus and cytoplasm separation. Although several previous articles have shown that some miRNAs act within the nucleus as activating RNAs [30–33], there have been no reports on RNAa facilitating tumour drug resistance. Herein, we identified differentially expressed miRNAs in nuclear RNA, cytoplasmic RNA, and total cell RNA in CAL27 and CAL27_CTX cells. RNA samples were reverse transcribed and tested by quantitative real-time fluorescent polymerase chain reaction (qRT‒PCR) to verify the nucleus-cytoplasm fractionation process. β-Actin and BIRC5 were used as cytoplasmic markers and were therefore enriched in the cytoplasm of both CAL27 and CAL27_CTX cells. U6, MALAT, and TUG1 were used as nuclear markers and therefore showed enrichment in the nucleus (Fig. S3a and b). The successfully fractioned RNA samples, along with total RNA samples from both cell lines, were then sent for small RNA sequencing.

**Fig. 1:**
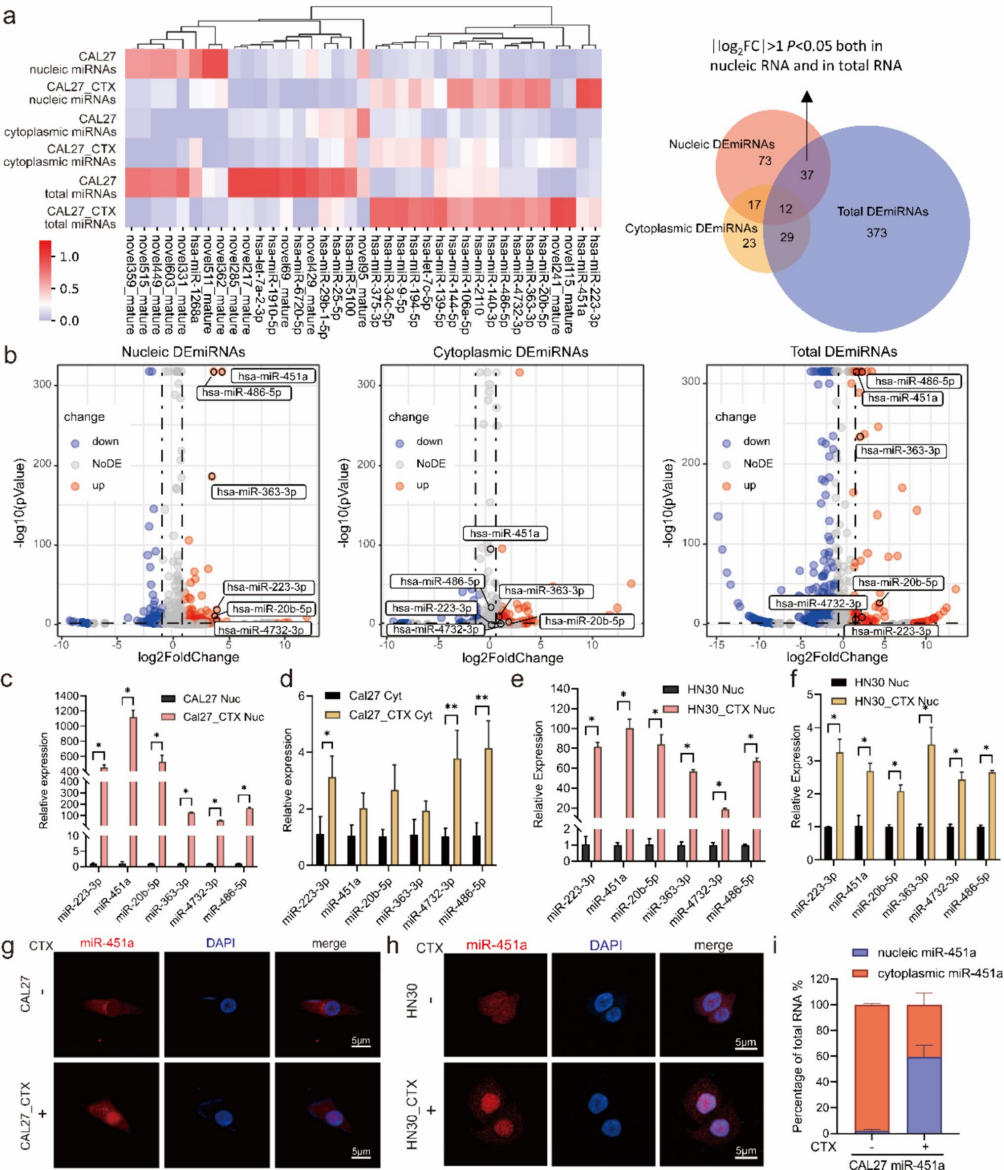
miR-451a was significantly enriched in the nucleus of CAL27_CTX cells (**a**) Small RNA sequences for nucleic miRNAs, cytoplasmic miRNAs and total miRNAs in CAL27 and CAL27_CTX cells. The numbers of differentially expressed miRNAs in nucleic, cytoplasmic and total CAL27_CTX cell samples are shown in the right panel. There were 37 differentially expressed miRNAs (DEmiRNAs) in both total and nucleic RNA. (**b**) DEmiRNAs in nucleic, cytoplasmic and total RNA samples. (**c–f**) qRT‒PCR analysis of the top enriched nuclear miRNAs. miRNAs in the nucleus of CAL27 and CAL27_CTX (**c**) and cytoplasm of CAL27 and CAL27_CTX (**d**), miRNAs in the nucleus of HN30 and HN30_CTX (**e**) and cytoplasm of HN30 and HN30_CTX (**f**). (**g**) FISH analysis of miR-451a in CAL27 and CAL27_CTX cells. (**h**) FISH analysis of miR-451a in HN30 and HN30_CTX cells. (**j**) qRT‒PCR analysis of the subcellular distribution of miR-451a in CAL27 miR-451a cells treated with or without cetuximab after nuclear-cytoplasmic separation. The data are presented as the means ± SDs. Student’s *t* test was performed (**c**–**f**; n = 3). (*P < 0.05, **P < 0.01, ***P < 0.001, ****P < 0.0001)

We focused on the group of miRNAs that were specifically differentially expressed in the nucleus but not in the cytoplasm. We excluded those miRNAs that were mainly differentially expressed in the cytoplasm and total lysates, which left the differentially expressed miRNAs in the nucleus and in the whole cells. Thirty-seven differentially expressed miRNAs in the nucleus and cytoplasm are shown (Fig. 1a, b). There were eight downregulated and twenty-nine upregulated genes in the nucleus, among which eighteen were upregulated in both the nucleus and total cells. The logarithm of the fold change and P value of differentially expressed miRNAs in the nucleus, cytoplasm and total cell lysates are shown (Fig. 1c–e). According to these results, the top 6 enriched miRNAs that were highly expressed in the CAL27_CTX nucleus and CAL27_CTX total RNA are marked. These genes are hsa-miR-451a, hsa-miR-486-5p, hsa-miR-363-3p, hsa-miR-223-3p, hsa-miR-20b-5p, and hsa-miR-4732-3p. qRT‒PCR was also performed to determine the relative expression of the miRNAs that were highly expressed in the nucleus of the cetuximab-resistant cell lines. The qRT‒PCR results showed that miR-451a was the most enriched in the CAL27_CTX nucleus. Its expression level was 1100 times higher than that in the nucleus of CAL27 cells (Fig. 1c, d). miR-451a was also markedly enriched in the HN30_CTX nucleus compared to the HN30 nucleus (Fig. 1e, f). Nuclear-cytoplasmic separation in HN30 and HN30_CTX were also verified by detecting the expression of markers via qRT‒PCR (Fig. S3c and d). The expression level of nuclear miR-451a also increased in CAL27 and HN30 after 48 h of treatment with cetuximab (Fig. S3e), suggesting that its enrichment inducible by cetuximab treatment. Fluorescence in situ hybridization (FISH) was performed to visualize the subcellular localization and nuclear enrichment of miR-451a. The results showed that the number of miR-451a clusters in the nucleus was significantly greater after cetuximab treatment in CAL27_CTX and HN30_CTX cells than in CAL27 and HN30 cells (Fig. 1g, h), as the fluorescence intensity of the miR-451a probe was greatly enhanced in the nucleus, but the intensity of the DAPI was not increased (Fig. S3f).

To further study the function of the upregulated miR-451a, we established a miR-451a-overexpressing cell strain through lentivirus transduction (referred to as CAL27 miR-451a and HN30 miR-451a hereafter) for future experiments and performed FISH and qRT‒PCR to validate miR-451a nuclear enrichment with and without cetuximab treatment. Intriguingly, as the results showed, only the cetuximab-treated miR-451a-overexpressing cells were significantly enriched. However, miR-451a was mostly located in the vast cytoplasm of cells that were not treated with cetuximab (Fig. 1i and Fig. S3g). Nuclear-cytoplasmic separation for miR-451a quantification was also validated via qRT‒PCR (Fig. S3h).

These findings suggested that several miRNAs significantly enriched in CAL27_CTX cells may be related to cetuximab resistance in these cells. The expression of miR-451a, which is among the significantly enriched nuclear miRNAs, was upregulated to the greatest extent, indicating the probable regulatory effect of miR-451a in the nucleus. Since this enrichment was closely related to cetuximab treatment, miR-451a is speculated to contribute to cetuximab resistance.

### Intranuclear miR-451a targets the KDM7A intron

**Fig. 2:**
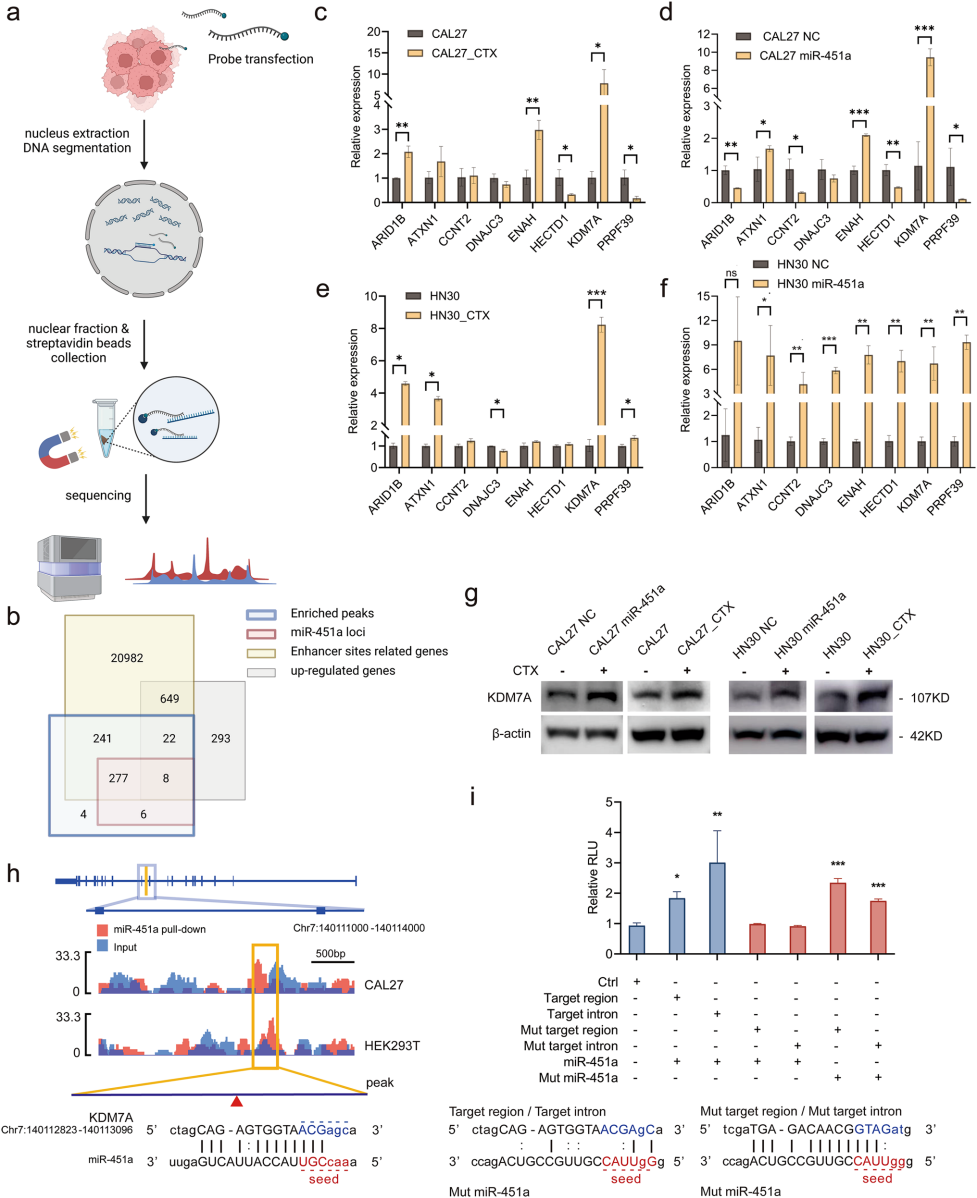
miR-451a interacts with an enhancer region in KDM7A and promotes KDM7A expression. (**a**) A schematic diagram of ChIRP-seq. (**b**) The filtering process for miR-451a nuclear targets. The ChIRP-seq results revealed differentially enriched peaks that are potential targets of miR-451a. Bioinformatics analysis with miRanda helped identify the miR-451a binding sites through score rating and energy calculations. ROSE was used to identify the enhancer region that contains the DE peaks. A microarray was used to identify the genes upregulated in the CAL27 miR-451a and CAL27_CTX cells (Figure S3a-c). Eight genes were eventually chosen after the screen. (**c–f**) qRT‒PCR analysis of the relative expression of the eight candidate miR-451a targets in CAL27_CTX and CAL27 (**c**), CAL27_CTX and CAL27 (**d**), HN30 miR-451a and HN30 NC (**e**), and HN30_CTX and HN30 (**f**) cells. (**g**) Western blot analysis showed upregulated KDM7A expression in CAL27_CTX and HN30_CTX cells. (**h**) miRanda analysis presented a binding site for miR-451a on the tenth KDM7A intron. The red arrow shows the specific binding site for miR-451a and the sequence. (**i**) A dual luciferase reporter assay was performed to examine the effect of the enhancer in the miR-451a binding region of KDM7A and the enhancer-located intron. The target region, intron and empty vector were cotransfected with miR-451a or the scrambled negative control. The mutated target region and intron were cotransfected with mutated but matched miR-451a. The data are presented as the means ± SDs. Student’s *t* test was performed. (c-f and i; n = 3) (*P < 0.05, **P < 0.01, ***P < 0.001, ***P < 0.0001)

To investigate the role of nuclear-enriched miR-451a, its nuclear target and cellular functions were investigated. Chromatin isolation by RNA purification and sequencing (ChIRP-seq) was performed to investigate the interaction between chromatin and intranuclear miR-451a. Biotin-labelled miR-451a probes were transfected into CAL27_CTX and HEK-293 T cells prior to the ChIRP assay. The experimental process modified from previous works[33, 34] is described in the diagram, and the obtained purified RNA-chromatin samples were subjected to ChIRP sequencing (Fig. 2a).

The sequencing results were analysed and filtered with results from four other parts of the analysis (Fig. 2b). The ChIRP sequencing results indicated that the most enriched peaks corresponded to miR-451a conjugates in the CAL27_CTX and 293 T cells. 293 T cells served as a control to help rule out nonspecific binding sites. The peaks enriched in these two cell lines were collected as common peaks and considered potential targets of miR-451a. Next, miRanda was used to score the binding probabilities for miR-451a and these potential targets, with a score threshold of 100. The results are provided in the supplementary material (file z_Result). Rank Ordering of Super-Enhancers (ROSE) was used to identify the enhancers and superenhancers within our target peaks and to identify the related genes. The results of the ROSE analysis are provided in the supplementary materials (file ROSE_result). As a result, 291 genes were considered to contain possible binding loci for miR-451a. A total of 21,894 genes were identified as enhancer-related genes, among which 285 genes intersected with the miR-451a-binding loci. In this way, we excluded only ten genes. This means that the peaks that scored highly as potential miR-451a targets were mostly enhancer related. Therefore, further investigation is required to narrow the candidates.

We performed a microarray on CAL27 and CAL27_CTX cells, in which miR-451a was overexpressed CAL27 (CAL27 miR-451a) and the counterpart scrambled negative control (referred to as CAL27 NC). The microarray results were compared between CAL27_CTX and CAL27 cells and between CAL27 miR-451a and CAL27 NC cells (Fig. S4a, b). The differentially expressed genes that intersect in both comparisons are provided in the supplementary material (file intersection up and intersection down). The 1145 genes that were upregulated in both CAL27_CTX and CAL27 miR-451a cells were selected for intersection with the previous results (Fig. S4c). PCA revealed more similarities between CAL27_CTX and CAL27 miR-451a than between the other two samples (Fig. S4d), which suggested that the cellular function of miR-451a may resemble the resistance observed in CAL27_CTX cells upon cetuximab stimulation. GO and KEGG analyses showed that the upregulated gene-enriched pathways were closely related to nuclear activating miRNAs and cetuximab resistance (Fig. S5).

According to the enrichment analysis, we successfully identified eight genes as potential miR-451a targets that are both enhancer related and were upregulated in the CAL27 miR-451a and CAL27_CTX samples. To confirm these results from the microarray assay and the bioinformatics analysis, we performed qRT‒PCR and compared the results from the same groups used for the microarray. Among the eight potential targets, KDM7A was most strongly enriched in the comparisons between CAL27_CTX and CAL27, between CAL27 and miR-451a and between CAL27 and NC, and between HN30_CTX and HN30 (Fig. 2c–e). Although KDM7A was not most enriched in HN30 miR-451a, it was still significantly upregulated (Fig. 2f). Western blot analysis also confirmed that KDM7A expression was greater in CAL27_CTX cells than in CAL27 cells and in CAL27 miR-451a cells (Fig. 2g). The same results were also observed in the HN30_CTX and HN30 miR-451a cells (Fig. 2g). When combined with the previous results, only one of the enriched peaks identified by ChIRP fell within the KDM7A sequence. This peak is located at the tenth intron of KDM7A. miRanda analysis showed a binding site (Fig. 2h).

To validate the binding site we identified, we synthesized a pGL3 luciferase reporter vector for the target peak region (target region) and the intron containing the region (target intron). A dual luciferase reporter assay showed that compared with transfection with the scramble NC, transfection with miR-451a significantly increased the RLUs of the target region and target intron reporter (Fig. 2i). Moreover, mutant miR-451a, which did not match the vectors, impaired this increase. However, cotransfection of the mutant miR-451a and mutant vectors that matched the mutant miR-451a restored the increase in RLUs (Fig. 2i).

Thus, we identified a target of nucleic miR-451a through strict filtering rules. We verified that miR-451a promotes KDM7A expression by targeting a binding site with an enhancer effect at the tenth intron of KDM7A. Thereafter, we explored the functions of miR-451a and KDM7A and their roles in cetuximab resistance.

### miR-451a promotes cetuximab resistance in vitro and in vivo

**Fig. 3:**
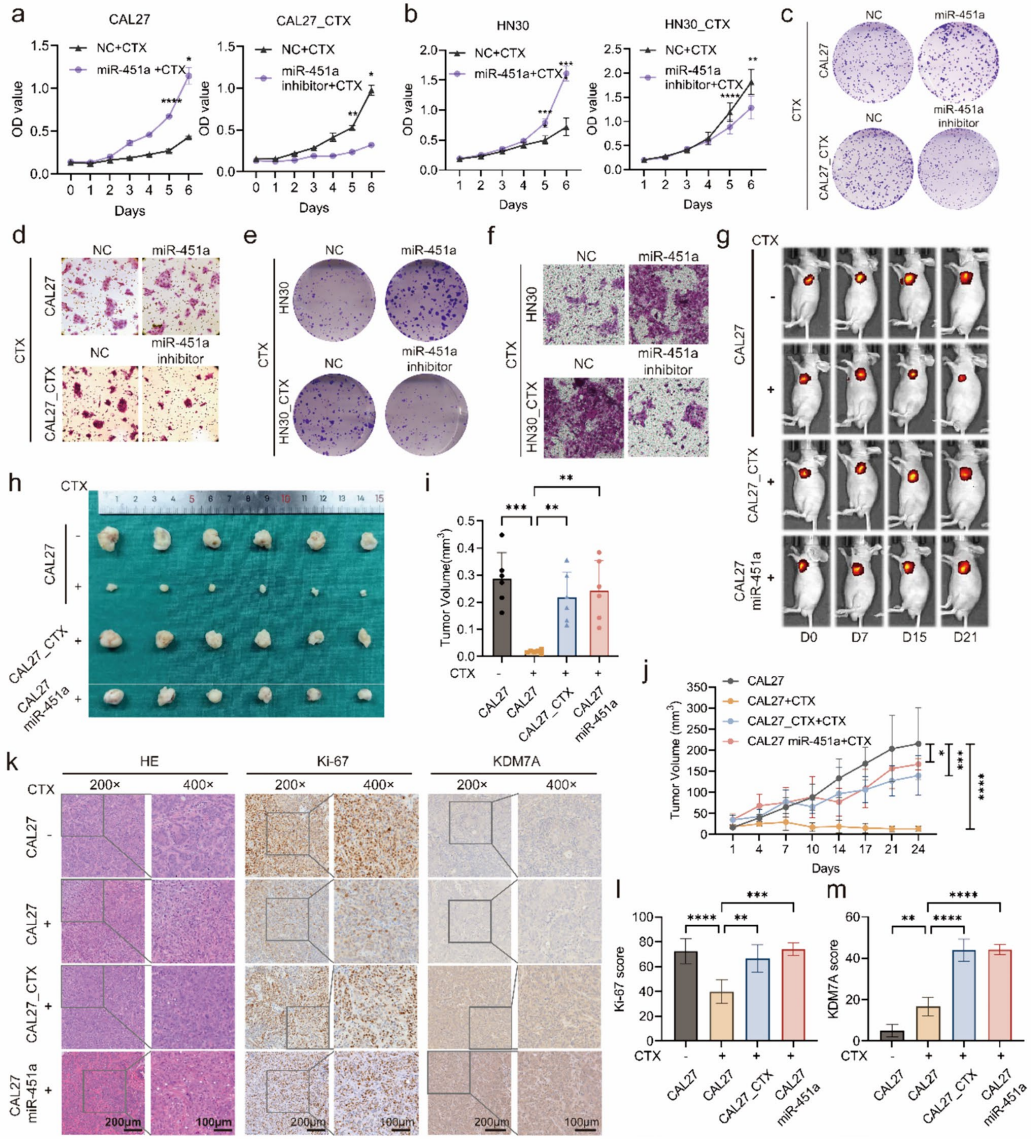
miR-451a enhanced CAL27 cell proliferation, migration and tumour growth upon treatment with cetuximab. (**a, b**) Growth curves of miR-451a-overexpressing CAL27 (**a** left) and HN30 (**b** left) cells and of miR-451a-knockdown CAL27_CTX (a right) and HN30_CTX (b right) cells and their corresponding negative control cells. (**c**–**f**) Colony formation and Transwell assays of miR-451a-overexpressing CAL27 (**c**, **d**) and HN30 cells (**e**, **f**), miR-451a-knockdown CAL27_CTX (**c**, **d**) and HN30_CTX cells (**e**, **f**) and their corresponding negative control cells. (**g–j**) In vivo experiments via subcutaneous tumour formation (**g** and **h**) and measurements of tumour growth (**j**) and tumour weight (**i**) in each group. (**k–m**) H&E and Ki-67 staining and KDM7A IHC results (**k**), the staining score for Ki-67 (**l**) and KDM7A staining (**m**). The data are presented as the means ± SDs of experiments conducted in triplicate. Two-way ANOVA was performed followed by Tukey’s test (**a**, **b** and **j**; n = 3 (**a**, **b**) and n = 6 (**j**)).. One-way ANOVA was performed followed by Tukey’s test (**i**, **l** and **m**; n = 6 (i), n = 5 (**l** and **m**)) (****P < 0.0001, **P < 0.01, *P < 0.05). “ns” is representative of no significance)

To investigate the role of miR-451a in cetuximab resistance, cell proliferation was evaluated by growth curve and colony formation assays. Compared with CAL27 and HN30 cells, CAL27 miR-451a and HN30 miR-451a cells treated with cetuximab exhibited an enhanced proliferation rate and increased colony number (Fig. 3a, b, c, e, Fig. S6a and c). In contrast, CAL27_CTX and HN30_CTX cells transfected with the miR-451a inhibitor exhibited a reduced proliferation rate and colony number (Fig. 3a, b, c, e, Fig. S6a and c). The Transwell and wound healing assays showed results similar to those of the growth curve and colony formation assays. The overexpression of miR-451a significantly increased the migration of CAL27 cells treated with 300 μg/mL cetuximab (Fig. 3d, f, Fig. S6b, d, e–g). In addition, miR-451a inhibition reduced CAL27_CTX and HN30_CTX cells migration.

In vivo experiments were carried out using a subcutaneous BALB/C nude mouse model. Tumour volumes were recorded via in vivo bioluminescence imaging and growth curves (Fig. 3g, j). Cetuximab administration began on day seven and proceeded every three days thereafter at a dose of 1 mg/mouse. At the end of the experimental period, the tumours in the CAL27 group grew the largest among the four groups (Fig. 3h). In contrast, when CAL27 tumours were given cetuximab, the tumours shrank drastically, yet CAL27_CTX tumours remained relatively large even with cetuximab treatment. Tumour volumes in the CAL27 miR-451a group treated with cetuximab were similar to those in the CAL27_CTX group at the end of the experiment. Tumour weight was ranked according to tumour growth (Fig. 3i). Similarly, subcutaneous tumours in the CAL27_CTX + antiago-miR-451a group exhibited reduced tumour volume and weight compared with those in the CAL27_CTX group (Fig. S6h-j). Cetuximab treatment markedly reduced the tumour weight in the CAL27 group compared to that in the other three groups. The tumours were sectioned, and H&E staining, Ki-67 staining, and KDM7A immunohistochemistry (IHC) were performed (Fig. 3k). The Ki-67 staining results were consistent with the cell growth trends (Fig. 3l). The CAL27 control group, CAL27_CTX group (cetuximab treated), and CAL27 miR-451a group (cetuximab treated) had significantly greater Ki-67 scores than did the CAL27 group (cetuximab treated). KDM7A staining in the CAL27 cells treated with cetuximab was slightly greater than that in the CAL27 control cells (Fig. 3m). The miR-451a levels in the CAL27_CTX and CAL27 groups were significantly higher than those in the CAL27 and CAL27 with cetuximab treatment groups, indicating that KDM7A was upregulated in these two groups.

In short, we validated that miR-451a promotes cetuximab resistance through in vitro and in vivo experiments. miR-451a promoted cell proliferation and migration and promoted tumour growth under cetuximab treatment conditions.

### KDM7A promotes cetuximab resistance in vitro and in vivo

**Fig. 4:**
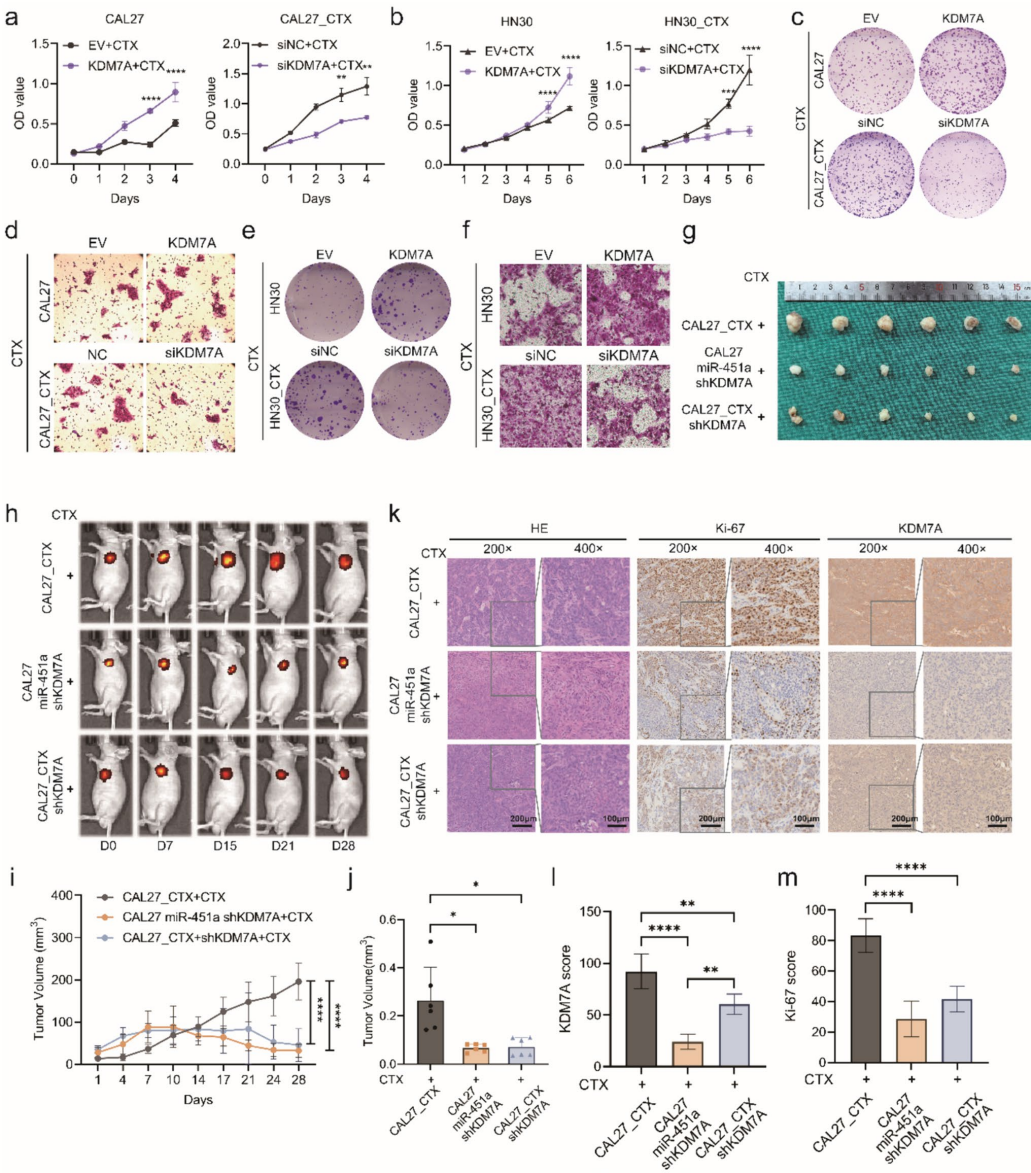
KDM7A promoted cell proliferation, migration and tumour growth upon treatment with cetuximab. (**a, b**) Growth curves of KDM7A-overexpressing CAL27 (**a**) and HN30 cells (**b**) and of KDM7A-knockdown CAL27_CTX (**a**) and HN30_CTX (**b**) cells. (**c–f**) Colony formation assays (**c** and **e**) and transwell assays (**d** and **f**) of KDM7A-overexpressing CAL27 and HN30 cells and KDM7A-knockdown CAL27_CTX and HN30_CTX cells. (**g–j**) Subcutaneous tumour formation using KDM7A knockdown CAL27_CTX and CAL27 miR-451a treated with cetuximab (**g** and **h**) and measurements of tumour growth (**i**) and tumour weight (**j**) in each group. (**k–m**) H&E and Ki-67 staining and KDM7A IHC results (**k**), the staining score for KDM7A staining (**l**) and Ki-67 (**m**). The data are presented as the means ± SDs. Two-way ANOVA was performed followed by Tukey’s test (**a**, **b** and **i**; n = 3 (**a** and **b**) and n = 6(**i**)). One-way ANOVA was performed followed by Tukey’s test (**j**, **l** and **m**; n = 6 (**j**), n = 5 (**l** and **m**)) (****P < 0.0001, ***P < 0.001, **P < 0.01, *P < 0.05). “ns” is representative of no significance)

As miR-451a was validated to promote cetuximab resistance in CAL27 and HN30 cells, we hypothesized that the target gene KDM7A would play the same role. We used siRNAs and overexpression vectors to knock down and overexpress KDM7A, respectively (Fig. S7a-b). Both growth curve and colony formation assays showed that KDM7A knockdown impaired CAL27_CTX and HN30_CTX cell proliferation and migration and that overexpressing KDM7A promoted CAL27 and HN30 cell proliferation and migration in response to cetuximab treatment (Fig. 4a–f, Fig. S7c-h). These in vitro results suggest that KDM7A facilitates cetuximab resistance in both CAL27 and HN30 cells at the cellular level.

shRNA was used to establish a KDM7A knockdown cell line as an in vivo model (Fig. S7i). Since we previously reported that CAL27 miR-451a cells are resistant to cetuximab, we used the shKDM7A vector to knock down KDM7A in CAL27 miR-451a and CAL27_CTX cells to determine whether cetuximab resistance was weakened. In vivo experiments revealed that compared with those in the CAL27_CTX group, both the CAL27 miR-451a shKDM7A and CAL27_CTX shKDM7A groups exhibited decreased tumour volumes (Fig. 4g–i) and tumour weights (Fig. 4j). All three groups were treated with cetuximab at the same dose used in the previous experiment. Similarly, the CAL27_CTX group had the highest Ki-67 and KDM7A IHC scores, and both of the other groups had distinctly lighter Ki-67 and KDM7A staining (Fig. 4k–m).

These results indicated that the miR-451a target gene KDM7A also enhances cetuximab resistance. Reduced KDM7A expression decreased the resistance of CAL27_CTX cells to cetuximab and decreased cell proliferation, migration and tumour growth.

### miR-451a-activated KDM7A expression is mediated by AGO2

**Fig. 5:**
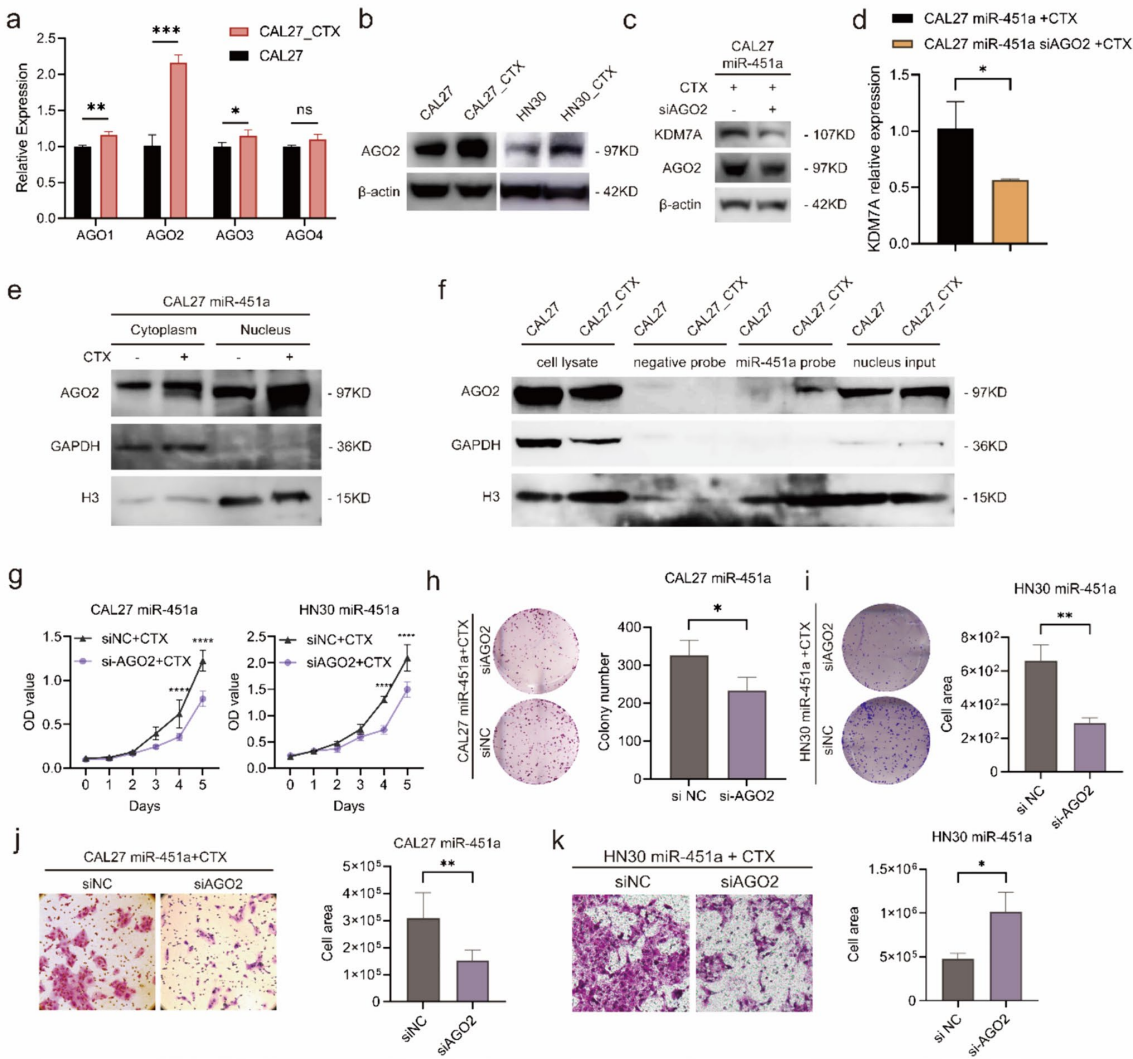
miR-451a enhanced KDM7A expression in an AGO2-dependent manner. (**a**) AGO2 was significantly upregulated among members of the AGO family in CAL27_CTX cells. (**b**) Western blot analysis showed higher AGO2 expression in CAL27_CTX and HN30_CTX cells. (**c, d**) Western blot (**c**) and qRT‒PCR (**d**) results showing that KDM7A expression decreased as AGO2 was knocked down in CAL27 miR-451a cells treated with cetuximab. (**e**) Western blot after nuclear-cytoplasmic separation showing the distribution of AGO2 in the nucleus and cytoplasm in CAL27 miR-451a cells. (**f**) RNA pulldown using miR-451a probe and western blot were performed to validate the involvement of AGO2 in the process of nuclear miR-451a activation. (**g–i**) Cell growth curve (**g**) and colony number (**h** and **i**) assays were performed after AGO2 was knocked down by siRNAs in CAL27 miR-451a and HN30 miR-451a cells. (**j, k**) Transwell assays were performed in AGO2-knockdown CAL27 miR-451a and HN30 miR-451a cells treated with cetuximab. The data are presented as the means ± SDs of experiments conducted in triplicate. Student’s *t* test was performed (**a**, **d**, **h**–**k**; n = 3 (**a**, **d**), n = 5 (**h**–**k**)). Two-way ANOVA was performed followed by Tukey’s test (**g**; n = 3). (****P < 0.0001, ***P < 0.001, **P < 0.01, *P < 0.05)

In the previous sections, we recognized miR-451a and its nuclear target gene KDM7A as both cetuximab resistance motivators in CAL27_CTX cells, but the factors that induce miRNA activation remain unclear. As nucleic-activating RNAs have been reported by researchers, several studies have suggested that the key factor that mediates this process is AGO2 [30, 33, 35–37], the same factor that mediates the RNA interference process. The function of AGO2 is not limited to RNA silencing. Evidence has shown that AGO also plays a vital role in the nucleus, as it interacts with chromatin and modulates its topological structure [38]. Since chromatin remodelling is an early event in the development of cetuximab resistance [39], we surmised that members of the AGO family are also closely associated with this process. In this study, we detected the expression levels of all four members of the human AGO family. The qRT‒PCR results showed that AGO2 was the most significantly upregulated protein in CAL27_CTX cells compared to that in CAL27 cells (Fig. 5a). Western blot analysis confirmed its upregulation in the cetuximab-resistant cell samples (Fig. 5b).

To confirm the function of AGO2 in miR-451a activation, we knocked down AGO2 through siRNA interference and found reduced KDM7A expression in CAL27_CTX cells at both the mRNA and protein levels (Fig. 5c, d and Fig. S7a). Nuclear-cytoplasmic separation was also conducted in CAL27 miR-451a cells treated with or without cetuximab. AGO2 was expressed at higher levels in both the cytoplasm and nucleus and was especially enriched in the nucleus in cetuximab-treated cells (Fig. 5e). Furthermore, RNA pull-down assays and western blot were performed to investigate the interaction between miR-451a and AGO2 (Fig. 5f). CAL27 and CAL27_CTX cell lysates were fractioned into nuclear lysates as previously described for the ChIRP method. As the results showed, AGO2 was detected only in the miR-451a probe conjugates in the CAL27_CTX nuclear lysates and in cell lysate and nuclear inputs from both cell samples. These results indicate that AGO2 was expressed in both the CAL27 and CAL27_CTX nuclei but that it only interacted with miR-451a in the CAL27_CTX nuclei, where it mediates gene upregulation and hence cetuximab resistance in the cell.

We also established enhancer region knockout (KO) cell lines in 293 T and CAL27_CTX cells. First, a ChIP‒qPCR assay using an H3K27ac antibody was performed to validate the presence of the enhancer marker H3K27ac in the predicted enhancer region. The results showed that the target site was significantly enriched in 293 T, CAL27_CTX and HN30_CTX cells, indicating that the miR-451a-targeted KDM7A enhancer site is also marked by H3K27ac (Fig. S7b). Further validation using ChIP‒qPCR showed that miR-451a and AGO2 directly interact with the target site in the enhancer region. In specific, the fold enrichment in the 293 T KO ER and CAL27_CTX CAL27_CTX KO ER cells was significantly lower than that in the corresponding empty vector-transfected cells according to the ChIRP-qPCR assay using miR-451a probes (Fig. S7c). In addition, the KDM7A level was also reduced in these enhancer KO cells (Fig. S7d). A ChIP‒qPCR assay using an anti-AGO2 antibody showed that the fold enrichment of the target region was reduced in CAL27_CTX KO ER cells and increased in CAL27_CTX and HN30_CTX cells (Fig. S7e-f).

In vitro experiments to test cell proliferation and migration were performed to validate the role of AGO2 using CAL27 miR-451a and HN30 miR-451a cells. The proliferation of AGO2-knockdown cells decreased after cetuximab treatment, as shown by growth curve and colony formation assays (Fig. 5g–i). Similarly, cell migration by transwell and wound healing assays were reduced in CAL27 miR-451a and HN30 miR-451a cells with AGO2 knockdown after cetuximab treatment (Fig. 5j, k and Fig. S7g, h). These in vitro results indicated that loss of AGO2 function impaired the cetuximab resistance induced by nuclear miR-451a enrichment.

According to our results, the reduced resistance induced by AGO2 knockdown suggested that AGO2 also participates in the activation process. As we have learned from previous works, miR-451a maturation is independent of Dicer processing but dependent on AGO2 splicing [40–42]. This means that the AGO2 expression level might influence miR-451a production. Since the lentivirus we used to overexpress miR-451a was constructed with its mature form, AGO2 knockdown in our study did not decrease the miR-451a level. These results showed that AGO2 is a critical factor for miR-451a nuclear gene activation upon stimulation with cetuximab.

### miR-451a and KDM7A are critical contributors to cetuximab resistance in HNSCC patients

**Fig. 6:**
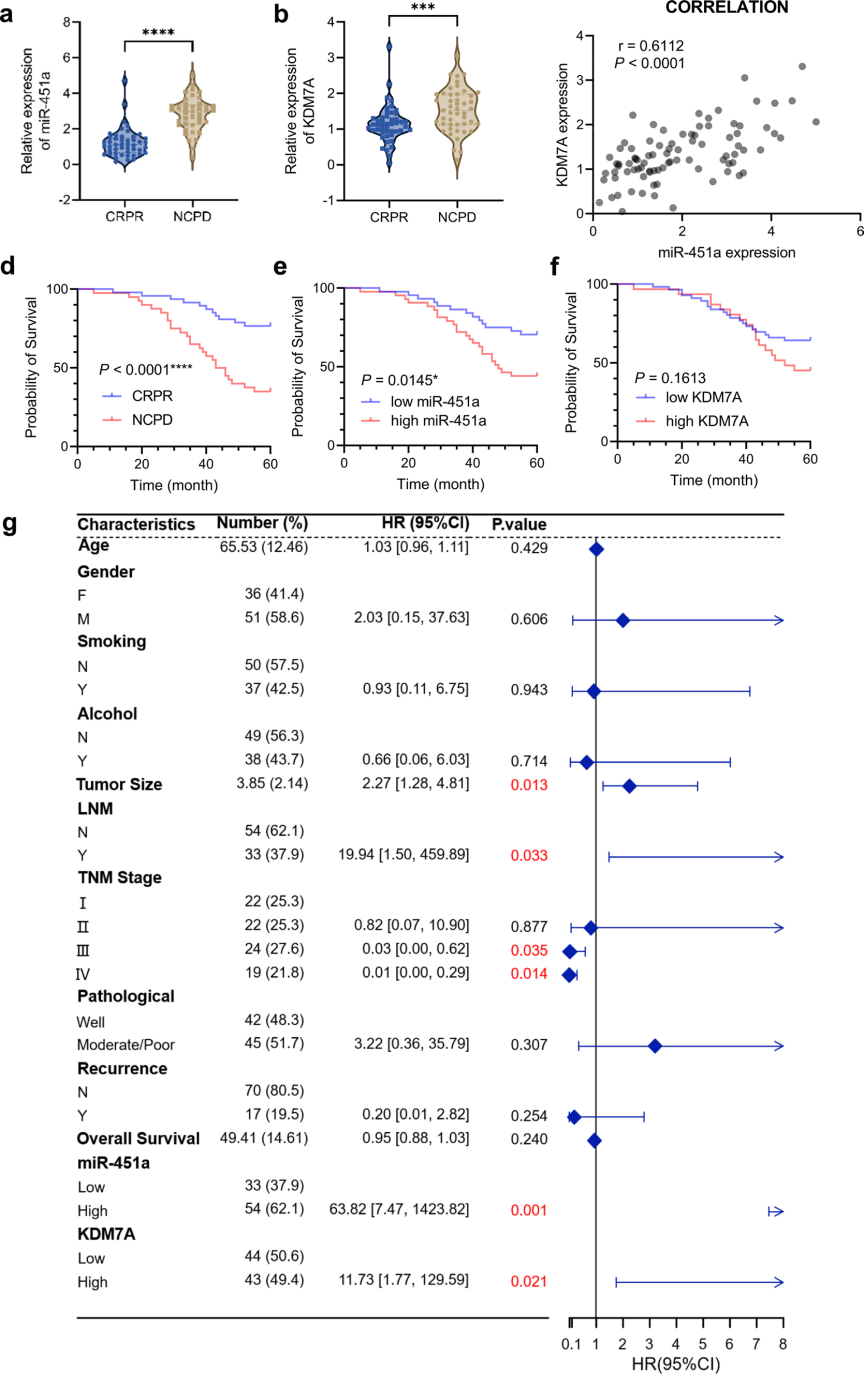
Assessment of clinical data showed that miR-451a and KDM7A are potent risk factors for cetuximab resistance. (**a**) Comparison of relative miR-451a expression level in the cetuximab-resistant group (NCPD) and that in the cetuximab-sensitive group (CRPR). (**b**) Comparison of relative KDM7A expression levels in the cetuximab-resistant group (NCPD) and that in the cetuximab-sensitive group (CRPR). (**c**) Pearson correlation analysis between miR-451a and KDM7A expression in HNSCC samples. (**d–f**) Survival analysis using the Kaplan‒Meier method between CRPR and NCPD group (**d**), low miR-451a and high miR-451a group (**e**) and low KDM7A and high KDM7A group (**f**).  (**g**) Multivariate logistic regression analysis revealed that tumour size, lymph node metastasis, and miR-451a and KDM7A expression levels are strong risk factors for cetuximab resistance. A higher TNM stage was considered a protective factor against cetuximab resistance. Student’s *t* test was performed (**a**, **b**). (****P < 0.0001, ***P < 0.001)

To further validate our results in patient samples showing that miR-451a and KDM7A are important contributors to cetuximab resistance and to explore the applicability of these two genes in cetuximab treatment and prognostics, we collected 87 patients with confirmed HNSCC who received cetuximab treatment from our centre. Among these patients, 47 had a complete response or partial response and were regarded as cetuximab sensitive. Forty patients were recorded as having no response or progressive disease and were regarded as having cetuximab resistance. qRT‒PCR was conducted to detect miR-451a and KDM7A expression. First, we compared the expression levels of miR-451a and KDM7A according to different clinicopathological features, including age, gender, smoking history, alcohol history, tumour size, lymph node metastasis, tumour-lymph node-metastasis (TNM) stage, pathological differentiation, efficacy of cetuximab, and recurrence (Table S4 and S5). Intriguingly, miR-451a and KDM7A expression differed exclusively in terms of the efficacy of cetuximab. In the cetuximab-resistant group (referred to as the NCPD group in the figure), the miR-451a and KDM7A levels were significantly greater than those in the cetuximab-sensitive group (referred to as the CRPR group in the figure) (Fig. 6a, b). The expression levels of KDM7A and miR-451a were strongly correlated in all the samples, with an r value of 0.6112, a 95% CI of 0.4598 to 0.7281 and a r^2^ of 0.3736. (Fig. 6c).

Overall survival analysis using the Kaplan‒Meier method showed a severely reduced survival rate in the NCPD group (Fig. 6d), indicating that cetuximab resistance shortened the survival time of patients. The miR-451a high-expression group also had a significantly lower survival rate than the miR-451a low-expression group (Fig. 6e), suggesting that high expression of miR-451a also impaired the survival rate of patients. Although a high expression level of KDM7A reduced the survival rate of patients who survived longer, it still had no significant effect on overall survival (Fig. 6f), possibly because of the limited sample size of the study.

Logistic regression was performed to analyse the following factors contributing to cetuximab resistance: age, sex, smoking history, alcohol consumption history, tumour size, lymph node metastasis status, TNM stage, pathological stage, recurrence, and especially miR-451a expression and KDM7A expression. The results showed that among all variables, tumour size, lymph node metastasis, TNM stages III and IV, high miR-451a expression and high KDM7A expression had significant impacts on cetuximab resistance (Fig. 6g). A larger tumour size, lymph node metastasis, high miR-451a expression and high KDM7A expression were recognized as risk factors with considerably high OR values.

These results showed that both miR-451a and KDM7A are significant risk factors for cetuximab resistance. Both high miR-451a expression and cetuximab resistance resulted in lower survival rates in HNSCC patients. In addition to high miR-451a and KDM7A expression, lymph node metastasis and tumour size are also risk factors for cetuximab resistance.

## Discussion

Squamous cell carcinoma of the head and neck region shares a high EGFR expression signature with carcinomas of other regions, such as colorectal cancer. However, intrinsic or acquired resistance to cetuximab or other EGFR inhibitors has decreased the response rate in patients, which has become a new challenge for physicians and cancer patients. To overcome such resistance to anti-EGFR therapies, new inhibitors have been developed, and new mechanisms that cause tumour resistance have been discovered.

Surprisingly, in HNSCC, the EGFR expression level is not a desirable marker for the outcome of combination treatment with cetuximab. Our results ruled out the impact of EGFR mutation on cultivated cetuximab resistance in our cell lines by exon sequencing. However, many studies have revealed downstream activation of the PI3K/AKT/mTOR, JAK-STAT, Wnt/β-catenin, and RAS/MAPK pathways, which are closely related to tumorigenesis and are responsible for cetuximab resistance [4, 16, 43–45]. The activation of PIK3CA, KRAS/HRAS, and STAT are key indicators of cetuximab resistance [46–49]. In an in vivo trial using cetuximab-resistant PDX models, researchers presented a series of biomarkers for intrinsic cetuximab resistance, including CDK1, ANKH, HEPHL1, MAPK15, SIX2, and PARP3. In addition, a decrease in the copy number of certain tumour suppressor genes that inhibit the previously mentioned pathways is also a promising candidate marker of cetuximab resistance. In particular, genetic dysfunction of PTEN, a negative regulator of the PI3K/AKT pathway, was suggested to contribute to cetuximab resistance in multiple studies [43, 44, 50, 51]. P53 is also widely acknowledged as a poor indicator for anticancer therapy [52, 53]. According to our microarray analysis, the pathways enriched in upregulated genes in the CAL27 miR-451a and CAL27_CTX cells were similar. We found p53 signalling pathway activation upon comparison of both sets of samples. Platinum drug resistance and EGFR tyrosine kinase inhibitor resistance pathways were activated in CAL27 miR-451a cells. The TNF signalling, Jak-STAT signalling, and Ras signalling pathways were activated in CAL27_CTX cells, consistent with previously reported findings. Hypothetically, cetuximab-EGFR interaction in resistant cell lines and the nuclear upregulated miR-451a altered the activity of the EGFR related pathways that impact the cellular response to EGFR-targeting drugs. However, the expression levels of these genes that are key nodes in these pathways have not been investigated thoroughly. According to the present results, miR-451a activation is strongly related to drug resistance and to the pathways that lead to it, indicating the potential value of nuclear miR-451a as a therapeutic target for treating cetuximab resistance.

Some epigenetic mechanisms of cetuximab resistance have been described in previous works. In CRC, the lncRNA MIR100HG has been demonstrated to activate the compensatory Wnt/β-catenin pathway through the regulation of miR-100 and miR-125b [16, 54]. The upregulated miRNAs target negative regulators of the Wnt/β-catenin signalling pathway and thereby activate this pathway indirectly, generating cetuximab resistance. Similarly, miR-320 was found to be sponged by circIFNGR2, which thus inhibits KRAS-induced cetuximab resistance in CRC [55]. miR-204 was shown to promote sensitivity to cetuximab in HNSCC cells by blocking the JAK-STAT signalling pathway. Recent studies concerning miRNAs that regulate cetuximab resistance have focused on a canonical mechanism, namely, posttranscriptional gene silencing via the RISC complex [56, 57]. Studies specific to the role of miR-451a have shown its dual potential in different cancers [58]. miR-451a promoted cell growth, migration, and EMT in osteosarcoma and activated the AKT/mTOR signalling pathway via YTHDC1-mediated m6A methylation [59]. In this study, nuclear miR-451a promoted cell growth under cetuximab stimulation, showing an onco-miRNA effect in HNSCC.

In this study, we focused on the functions of nuclear-enriched miRNAs that mediate HNSCC cetuximab resistance. Only a few previous reports have focused on the function of nuclear activating RNAs in disease regulation. The number of reports on nuclear miRNAs continues to increase as sequencing technologies mature. However, the mechanisms underlying these nuclear activation processes are incompletely understood. Some nuclear miRNAs are involved in the biogenic regulation of other noncoding RNAs. For instance, miR-10b binds U6 snRNA and modulates the methylation and pseudouridylation of U6, thus altering the RNA splicing machinery in glioblastoma [60]. Some miRNAs are nuclear-activating miRNAs that target promoter or enhancer regions. The enhancer targeting miR-492 activates NR2C1 expression and further enhances EMT in pancreatic cancer via the TGF-β/Smad3 pathway [61]. In another study, miR-195-5p targeted the FOXO3 promoter in an AGO2-dependent manner [35]. Additionally, the miRNA-AGO complex is guided into the nucleus by the RISC component TNRC6 [62, 63] and importin 8. [64, 65] In the present study, we clarified only the interaction between AGO2 and miR-451a and their roles in KDM7A activation and cetuximab resistance. Previous work has demonstrated that EGFR directly interacts with and activates AGO2, which further regulates miRNA processing and maturation [66]. EGFR activity is related to AGO2 function and mRNA nuclear enrichment [67]. Yet whether this process also mediates miRNA nuclear transport was not investigated in our current work. The other factors that interact with AGO2 and lead to KDM7A transcription were also not further explored and may be addressed in future works. Furthermore, the results of HNSCC patient data analysis also revealed the promising potential of miR-451a as a cetuximab resistance indicator. Taken together, these findings indicate that miR-451a has potential value as both a therapeutic target and predictive biomarker.

There are limited reports on the function of the histone demethylase KDM7A in tumorigenesis and drug resistance. KDM7A stimulates cell growth and migration and impairs cell death in bladder cancer. Further experiments showed that inhibiting KDM7A reduced the cisplatin resistance of bladder cancer cells [68]. Another study showed that KDM7A is related to enzalutamide resistance in prostate cancer [69], suggesting that KDM7A may be a prevalent marker for tumour drug resistance. Similarly, KDM7A was shown to be associated with stem cell maintenance in breast cancer and a reduction in apoptosis. BCL-2 was found to be positively related to KDM7A levels [70]. In our work, BCL-2 was also found to be highly expressed in CAL27 miR-451a and CAL27_CTX cells by microarray analysis. Considering the histone methyltransferase nature of KDM7A, the microarray results may indicate a potential correlation between KDM7A and the activated pathways, which requires more in-depth study in the future.

In summary, our study demonstrated a route of nuclear activation of miR-451a and AGO2-mediated cetuximab resistance in HNSCC. Cetuximab treatment was shown to be crucial for miR-451a nuclear localization. Herein, our results confirmed that cetuximab treatment promotes nuclear miR-451a-mediated activation of KDM7A expression by targeting an enhancer region in its intron. This miRNA‒DNA interaction is validated to be AGO2-dependent. Furthermore, analyses of HNSCC patient data showed that miR-451a and KDM7A are significant risk factors for cetuximab resistance in HNSCC, suggesting the great potential of miR-451a and KDM7A to serve as cetuximab resistance biomarkers or potential targets for overcoming cetuximab resistance in HNSCC. These promising results encouraged us to further investigate this nuclear activation mechanism in future works.

### Supplementary Information

Below is the link to the electronic supplementary material.

Supplementary file1 (PDF 1901 KB)

Supplementary file2 (XLSX 10 KB)

Supplementary file3 (XLSX 577 KB)

Supplementary file4 (XLSX 537 KB)

Supplementary file5 (XLSX 17145 KB)

Supplementary file6 (CSV 22 KB)

## Declarations

### Availability of data and material

The data that support the findings of this study are available within the article and its supplementary materials.

### Data availability statement

Microarray, small RNA sequencing and ChIRP-seq data generated from this study have been deposited in the Gene Expression Omnibus (GEO accession numbers GSE245159 and GSE244948 and GSE245153, respectively). Additional data are available from the corresponding author upon request.

## References

[1] Sung H, Ferlay J, Siegel RL (2021). Global cancer statistics 2020: GLOBOCAN estimates of incidence and mortality worldwide for 36 cancers in 185 countries. CA Cancer J Clin.

[2] Solomon B, Young RJ, Rischin D (2018). Head and neck squamous cell carcinoma: genomics and emerging biomarkers for immunomodulatory cancer treatments. Semin Cancer Biol.

[3] Pearson HE, Iida M, Orbuch RA (2018). Overcoming resistance to cetuximab with honokiol, a small-molecule polyphenol. Mol Cancer Ther.

[4] Burtness B, Bauman JE, Galloway T (2013). Novel targets in HPV-negative head and neck cancer: overcoming resistance to EGFR inhibition. Lancet Oncol.

[5] Johnson DE, Burtness B, Leemans CR (2020). Head and neck squamous cell carcinoma. Nat Rev Dis Prim.

[6] Bonner JA, Harari PM, Giralt J (2010). Radiotherapy plus cetuximab for locoregionally advanced head and neck cancer: 5-year survival data from a phase 3 randomizedrandomised trial, and relation between cetuximab-induced rash and survival. Lancet Oncol.

[7] Muraro E, Fanetti G, Lupato V (2021). Cetuximab in locally advanced head and neck squamous cell carcinoma: biological mechanisms involved in efficacy, toxicity and resistance. Crit Rev Oncol Hematol.

[8] Chen LF, Cohen EEW, Grandis JR (2010). New strategies in head and neck cancer: understanding resistance to epidermal growth factor receptor inhibitors. Clin Cancer Res.

[9] Leonard B, Brand TM, O’Keefe RA (2018). BET inhibition overcomes receptor tyrosine kinase-mediated cetuximab resistance in HNSCC. Cancer Res.

[10] Bardelli A, Siena S (2010). Molecular mechanisms of resistance to cetuximab and panitumumab in colorectal cancer. J Clin Oncol.

[11] Chung KY, Shia J, Kemeny NE (2005). Cetuximab shows activity in colorectal cancer patients with tumors that do not express the epidermal growth factor receptor by immunohistochemistry. J Clin Oncol.

[12] Saltz LB, Meropol NJ, Loehrer PJ (2004). Phase II trial of cetuximab in patients with refractory colorectal cancer that expresses the epidermal growth factor receptor. J Clin Oncol.

[13] Rabinowits G, Haddad RI (2012). Overcoming resistance to EGFR inhibitor in head and neck cancer: a review of the literature. Oral Oncol.

[14] Madoz-Gúrpide J, Zazo S, Chamizo C (2015). Activation of MET pathway predicts poor outcome to cetuximab in patients with recurrent or metastatic head and neck cancer. J Transl Med.

[15] Sun L, Fang Y, Wang X (2019). MiR-302a inhibits metastasis and cetuximab resistance in colorectal cancer by targeting NFIB and CD44. Theranostics.

[16] Lu Y, Zhao X, Liu Q (2017). LncRNA MIR100HG-derived miR-100 and miR-125b mediate cetuximab resistance via Wnt/β-catenin signaling. Nat Med.

[17] Liu J, Yang T, Huang Z (2022). Transcriptional regulation of nuclear miRNAs in tumorigenesis (review). Int J Mol Med.

[18] Bartel DP (2004). MicroRNAs: genomics, biogenesis, mechanism, and function. Cell.

[19] Pestova T, Kolupaeva V, Lomakin I (2007). Molecular mechanisms of translation initiation in eukaryotes. PNAS.

[20] Tay Y, Zhang J, Thomson AM (2008). MicroRNAs to Nanog, Oct4 and Sox2 coding regions modulate embryonic stem cell differentiation. Nature.

[21] Huang V, Long-Cheng L (2012). miRNA goes nuclear. RNA Biol.

[22] Younger ST, Corey DR (2011). Transcriptional gene silencing in mammalian cells by miRNA mimics that target gene promoters. Nucleic Acids Res.

[23] Place RF, Li LC, Pookot D (2018). MicroRNA-373 induces expression of genes with complementary promoter sequences. Proc Natl Acad Sci U S A.

[24] Xiao M, Li J, Li W (2017). MicroRNAs activate gene transcription epigenetically as an enhancer trigger. RNA Biol.

[25] Liang Y, Lu Q, Li W (2021). Reactivation of tumour suppressor in breast cancer by enhancer switching through NamiRNA network. Nucleic Acids Res.

[26] Liu H, Lei C, He Q (2018). Nuclear functions of mammalian MicroRNAs in gene regulation, immunity and cancer. Mol Cancer.

[27] Matsui M, Chu Y, Zhang H (2013). Promoter RNA links transcriptional regulation of inflammatory pathway genes. Nucleic Acids Res.

[28] Kang MR, Park KH, Yang JO (2016). miR-6734 Up-regulates p21 gene expression and induces cell cycle arrest and apoptosis in colon cancer cells. PLoS ONE.

[29] Qu H, Zheng L, Pu J (2015). miRNA-558 promotes tumorigenesis and aggressiveness of neuroblastoma cells through activating the transcription of heparanase. Hum Mol Genet.

[30] Li H, Fan J, Zhao Y (2019). Nuclear miR-320 mediates diabetes-induced cardiac dysfunction by activating transcription of fatty acid metabolic genes to cause lipotoxicity in the heart. Circ Res.

[31] Wang J, Huang V, Ye L (2014). Identification of small activating RNAs that enhance endogenous OCT4 expression. Stem Cells Dev.

[32] Janowski BA, Younger ST, Hardy DB (2007). Activating gene expression in mammalian cells with promoter-targeted duplex RNAs. Nat Chem Biol.

[33] Portnoy V, Lin SHS, Li KH (2016). SaRNA-guided Ago2 targets the RITA complex to promoters to stimulate transcription. Cell Res.

[34] Chu C, Qu K, Zhong FL (2011). Genomic maps of long noncoding rna occupancy reveal principles of RNA-chromatin interactions. Mol Cell.

[35] Bai Y, Pan B, Zhan X (2021). Microrna 195–5p targets foxo3 promoter region to regulate its expression in granulosa cells. Int J Mol Sci.

[36] Li H, Zhan J, Zhao Y (2020). Identification of ncRNA-mediated functions of nucleus-localized mir-320 in cardiomyocytes. Mol Ther Nucleic Acids.

[37] Li LC, Okino ST, Zhao H (2006). Small dsRNAs induce transcriptional activation in human cells. Proc Natl Acad Sci U S A.

[38] Nazer E, Gómez Acuña L, Kornblihtt AR (2022). Seeking the truth behind the myth: argonaute tales from “nuclearland”. Mol Cell.

[39] Kagohara LT, Zamuner F, Davis-Marcisak EF (2020). Integrated single-cell and bulk gene expression and ATAC-seq reveals heterogeneity and early changes in pathways associated with resistance to cetuximab in HNSCC-sensitive cell lines. Br J Cancer.

[40] Cheloufi S, Dos Santos CO, Chong MMW, Hannon GJ (2010). A dicer-independent miRNA biogenesis pathway that requires Ago catalysis. Nature.

[41] Kretov DA, Walawalkar IA, Mora-Martin A (2020). Ago2-dependent processing allows miR-451 to evade the global microRNA turnover elicited during erythropoiesis. Mol Cell.

[42] Kakumani PK, Ko Y, Ramakrishna S (2023). CSDE1 promotes miR-451 biogenesis. Nucleic Acids Res.

[43] Leblanc O, Vacher S, Lecerf C (2020). Biomarkers of cetuximab resistance in patients with head and neck squamous cell carcinoma. Cancer Biol Med.

[44] Gomes INF, da Silva-Oliveira RJ, da Silva LS (2022). Comprehensive molecular landscape of cetuximab resistance in head and neck cancer cell lines. Cells.

[45] Ju H, Hu Z, Lu Y (2020). TLR4 activation leads to anti-EGFR therapy resistance in HNSCC. Am J Cancer Res.

[46] Izumi H, Wang Z, Goto Y (2021). Pathway-specific genome editing of PI3K/mTOR tumor suppressor genes reveals that PTEN loss contributes to cetuximab resistance in head and neck cancer. Mol Cancer Ther.

[47] Rampias T, Giagini A, Siolos S (2014). RAS/PI3K crosstalk and cetuximab resistance in head and neck squamous cell carcinoma. Clin Cancer Res.

[48] Willey CD, Anderson JC, Trummell HQ (2019). Differential escape mechanisms in cetuximab-resistant head and neck cancer cells. Biochem Biophys Res Commun.

[49] Lièvre A, Bachet JB, Le Corre D (2006). KRAS mutation status is predictive of response to cetuximab therapy in colorectal cancer. Cancer Res.

[50] Izumi H, Wang Z, Goto Y (2020). Pathway-specific genome editing of PI3K / mTOR tumor suppressor genes reveals that PTEN loss contributes to cetuximab resistance in head and neck cancer. Mol Cancer Ther.

[51] Yao Y, Wang Y, Chen L (2022). Clinical utility of PDX cohorts to reveal biomarkers of intrinsic resistance and clonal architecture changes underlying acquired resistance to cetuximab in HNSCC. Signal Transduct Target Ther.

[52] Cutilli T, Leocata P, Dolo V, Altobelli E (2013). Evaluation of p53 protein as a prognostic factor for oral cancer surgery. Br J Oral Maxillofac Surg.

[53] López-Verdín S, Lavalle-Carrasco J, Carreón-Burciaga RG (2018). Molecular markers of anticancer drug resistance in head and neck squamous cell carcinoma: a literature review. Cancers (Basel).

[54] Thomas H (2017). MiR-100 and miR-125b induce cetuximab resistance in CRC. Nat Rev Gastroenterol Hepatol.

[55] Zhang Q, Zheng Y, Liu J (2023). CircIFNGR2 enhances proliferation and migration of CRC and induces cetuximab resistance by indirectly targeting KRAS via sponging to MiR-30b. Cell Death Dis.

[56] Yang S, Yuan ZJ, Zhu YH (2021). lncRNA PVT1 promotes cetuximab resistance of head and neck squamous cell carcinoma cells by inhibiting miR-124-3p. Head Neck.

[57] Citron F, Segatto I, Musco L (2021). miR-9 modulates and predicts the response to radiotherapy and EGFR inhibition in HNSCC. EMBO Mol Med.

[58] Khordadmehr M, Jigari-Asl F, Ezzati H (2019). A comprehensive review on miR-451: a promising cancer biomarker with therapeutic potential. J Cell Physiol.

[59] Cao D, Ge S, Li M (2022). MiR-451a promotes cell growth, migration and EMT in osteosarcoma by regulating YTHDC1-mediated m6A methylation to activate the AKT/mTOR signaling pathway. J Bone Oncol.

[60] El Fatimy R, Zhang Y, Deforzh E (2022). A nuclear function for an oncogenic microRNA as a modulator of snRNA and splicing. Mol Cancer.

[61] Liu S, He X, Di Y (2023). NamiRNA-enhancer network of miR-492 activates the NR2C1-TGF-β/Smad3 pathway to promote epithelial-mesenchymal transition of pancreatic cancer. Carcinogenesis.

[62] Nishi K, Nishi A, Nagasawa T (2013). Human TNRC6A is an Argonaute-navigator protein for microRNA-mediated gene silencing in the nucleus Human TNRC6A is an Argonaute-navigator protein for microRNA-mediated gene silencing in the nucleus. RNA.

[63] Katz S, Cussigh D, Urbán N (2016). A nuclear role for miR-9 and argonaute proteins in balancing quiescent and activated neural stem cell states. Cell Rep.

[64] Schraivogel D, Schindler SG, Danner J (2015). Importin-β facilitates nuclear import of human GW proteins and balances cytoplasmic gene silencing protein levels. Nucleic Acids Res.

[65] Wei Y, Li L, Wang D (2014). Importin 8 regulates the transport of mature microRNAs into the cell nucleus. J Biol Chem.

[66] Shen J, Xia W, Khotskaya YB (2013). EGFR modulates microRNA maturation in response to hypoxia through phosphorylation of AGO2. Nature.

[67] Dittmann K, Mayer C, Czemmel S (2017). New roles for nuclear EGFR in regulating the stability and translation of mRNAs associated with VEGF signaling. PLoS ONE.

[68] Lee K-H, Kim B-C, Jeong S-H (2020). Histone demethylase KDM7A regulates androgen receptor activity, and its chemical inhibitor TC-E 5002 overcomes cisplatin-resistance in bladder cancer cells. Int J Mol Sci Artic.

[69] Lee K-H, Hong S, Kang M (2018). Histone demethylase KDM7A controls androgen receptor activity and tumor growth in prostate cancer. Int J Cancer.

[70] Meng Z, Liu Y, Wang J (2020). Histone demethylase KDM7A is required for stem cell maintenance and apoptosis inhibition in breast cancer. J Cell Physiol.

