# Supplementary material for: Nuclear miR-451a activates KDM7A and leads to cetuximab resistance in head and neck squamous cell carcinoma
Source: Cell Mol Life Sci. 2024 Jun 28;81(1):282. doi: 10.1007/s00018-024-05324-x (PMC11335205; doi:10.1007/s00018-024-05324-x)
Supplement: Supplementary file 1 — Supplementary file1 (PDF 1901 KB) [file 18_2024_5324_MOESM1_ESM.pdf]

Supplementary Materials for

Nuclear miR-451a activates KDM7A and leads to cetuximab resistance in head and neck squamous cell carcinoma

Peisong Zhai, Tong Tong, Xiaoning Wang, Chuwen Li, Chun Liu, Xing Qin, Shu Li, Fei Xie, Jiayi Mao, Jianjun Zhang, Haiyan Guo

Corresponding Authors:

Jianjun Zhang

Department of Oral and Maxillofacial-Head & Neck Oncology, Shanghai Ninth People's Hospital, Shanghai Jiao Tong University School of Medicine, Shanghai 200011, PR China

Haiyan Guo

Department of Clinical Laboratory, Shanghai Ninth People's Hospital, Shanghai Jiao Tong University School of Medicine, Shanghai, 200011, PR China

Tel.: (86) 21-23271699-4239;

E-mail address: Haiyan Guo (sxguohaiyan@126.com) or Jianjun Zhang (zjjbio@sjtu.edu.cn).

Supplementary data: 8 figures, 5 tables, 5 spreadsheets

Supplemental figures

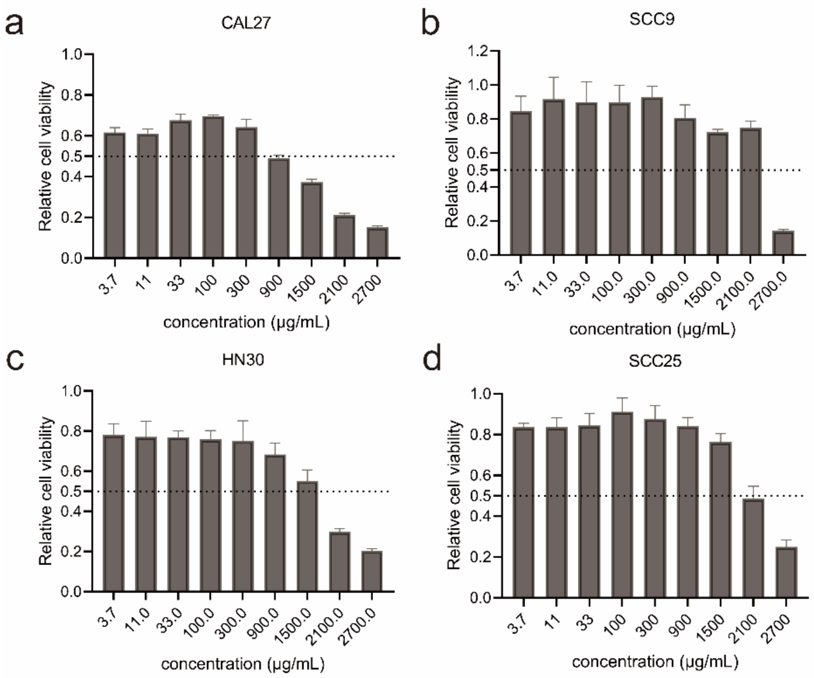

Figure S1. CAL27 and HN30 are relatively sensitive to cetuximab. Survival rates on HNSCC cells including CAL27 **(a)**, SCC9 **(b)**, HN30 **(c)** and SCC25 **(d)**, when treated with different concentrations of cetuximab.

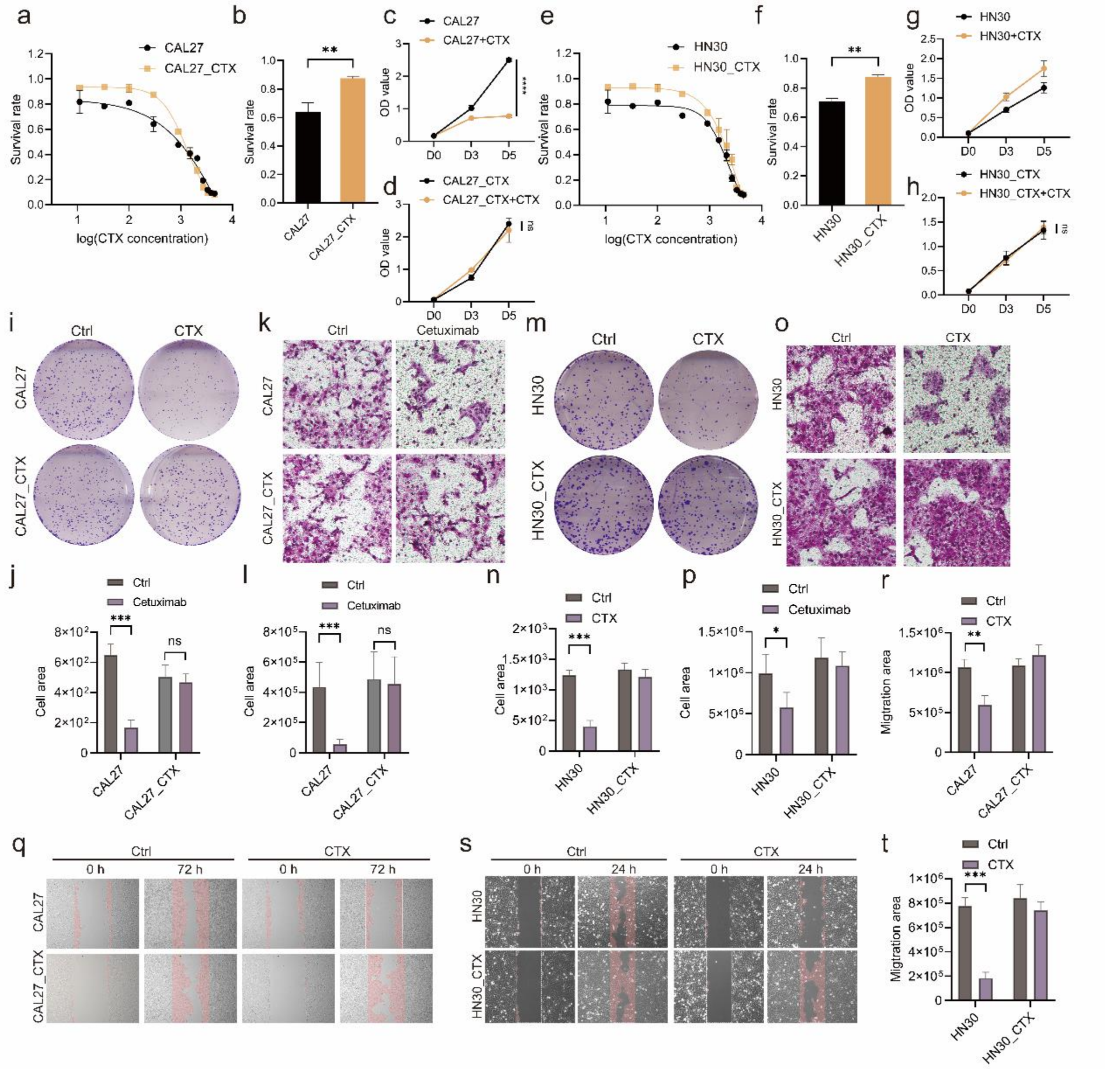

Figure S2. Cetuximab-resistant HNSCC cell lines establishment (a) The survival rates of CAL27 cells and CAL27_CTX cells. **(b)** The survival rates of CAL27 and CAL27_CTX cells treated with 300 μg/mL cetuximab. **(c-d)** Cell growth curves of CAL27 cells (c) and CAL27_CTX cells (d) with and without 5-day treatment of cetuximab. **(e)** Comparison of survival rates between HN30 and HN30_CTX. **(f)** The survival rates of HN30 and HN30_CTX cells treated with 300 μg/mL cetuximab. **(g)** Cell growth curves of HN30 cells (g) and HN30_CTX cells (h) with and without 5-day treatment of cetuximab. **(i-j)** Colony formation assays (i) and statistical analysis (j) of CAL27 and CAL27_CTX cells with and without cetuximab treatment. **(k-l)** Transwell assays (k) and statistical analysis (l) of CAL27 and CAL27_CTX cells with and without cetuximab treatment. **(m-n)** Colony formation assays (m) and statistical analysis (n) using HN30 and HN30_CTX cells with and without cetuximab treatment. **(o-p)** Transwell assays and statistical analysis using HN30 and HN30_CTX cells with and without cetuximab treatment. **(q-t)** Wound healing assay and statistical analyses using CAL27 and CAL27_CTX cells (q and r) and HN30 and HN30_CTX cells (s and t) with and without cetuximab treatment. T tests (b, f, j, l, n, p, r and t; n=3) and two-way ANOVA (c, d, g and h; n=3) were performed. ****P < 0.0001, ***P < 0.001, **P < 0.01, *P < 0.05; "ns" indicates no significance.

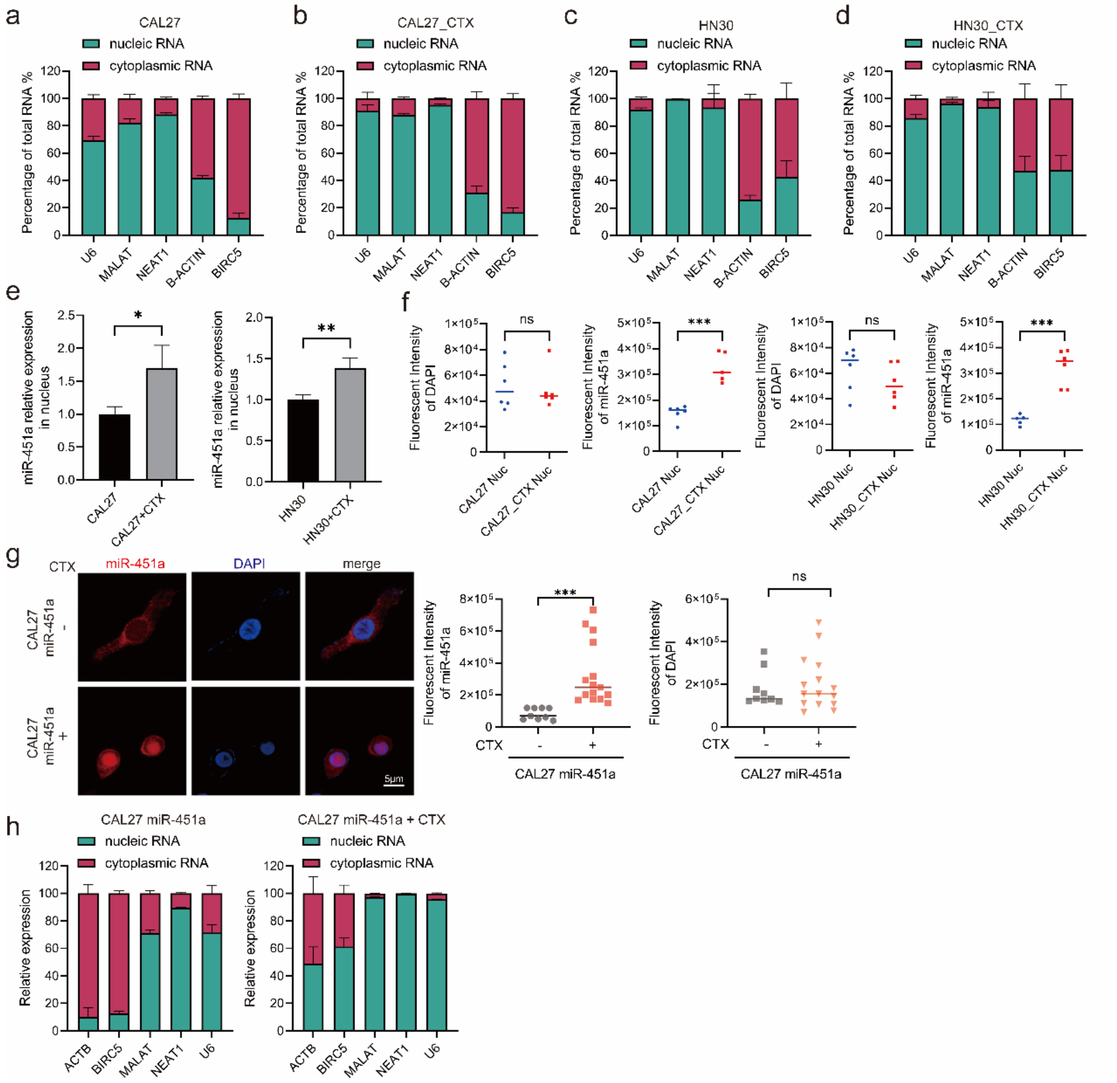

Figure S3. miR-451a expression increased in the nucleus of HNSCC cells treated with cetuximab. (a-d) Verification of the nuclear-cytoplasmic separation of CAL27 **(a)**, CAL27_CTX **(b)**, HN30 **(c)** and HN30_CTX **(d)** cells using qRTPCR. **(e)** The miR-451a expression level increased in nucleus of CAL27 and HN30 cells after 48 h of treatment with cetuximab. **(f)** The miR-451a fluorescence intensity in the CAL27 nucleus, CAL27_CTX nucleus, HN30 nucleus and HN30_CTX nucleus. **(g)** FISH analysis of miR-451a in CAL27 miR-451a cells with and without cetuximab treatment. **(h)** Nuclear-cytoplasmic separation verification by qRT–PCR analysis of miR-451a in CAL27 miR-451a cells treated with or without cetuximab. β-Actin and BIRC5 were mainly expressed in the nucleus. MALAT, NEAT1 and U6 were mainly expressed in the cytoplasm. T tests were performed (e, f and g). ****P < 0.0001, ***P < 0.001, **P < 0.01, *P < 0.05; "ns" indicates no significance.

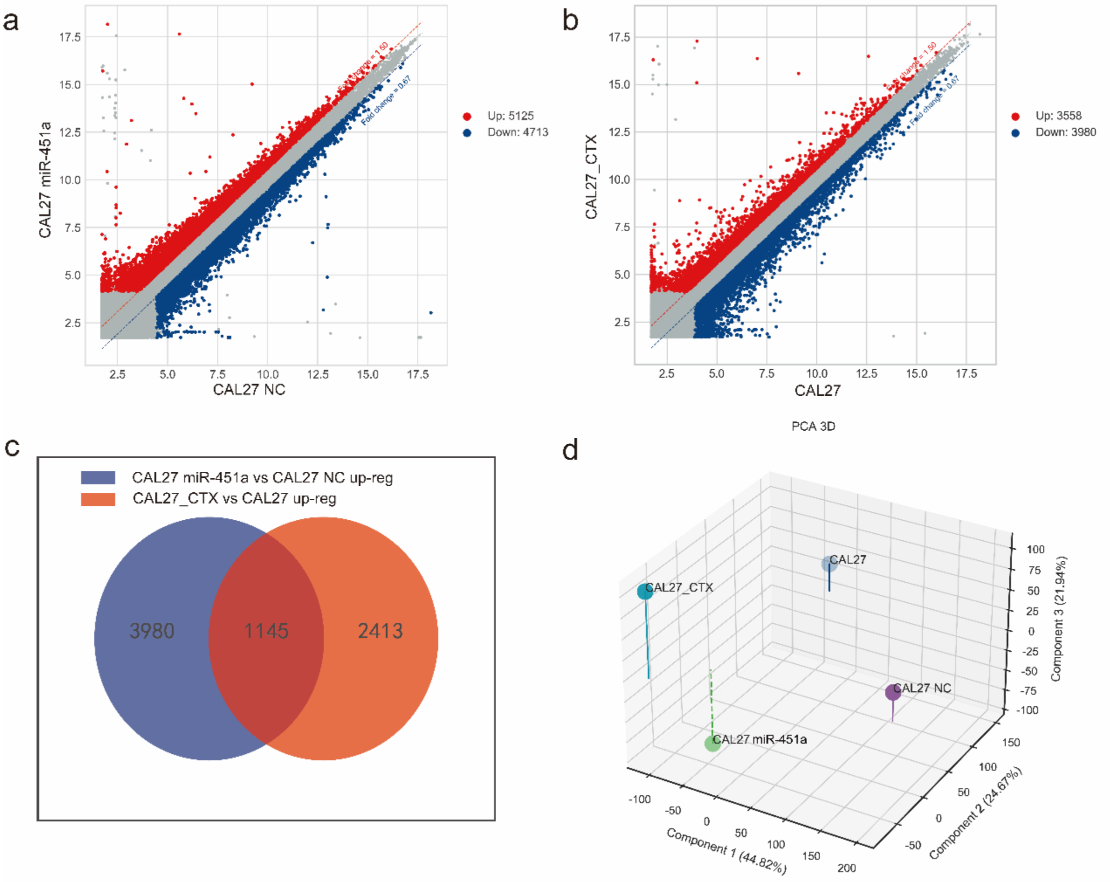

Figure S4. Microarray data analysis of CAL27 miR-451a, CAL27 NC, CAL27 and CAL27_CTX. **(a)** Volcano plot showing the DEGs in the CAL27 miR-451a group compared to the CAL27 NC group. There were 5125 upregulated genes and 4713 downregulated genes. **(b)** The volcano plot shows the DEGs in CAL27_CTX compared to those in CAL27. There were 3558 upregulated genes and 3980 downregulated genes. **(c)** The intersection of the upregulated genes in the two comparisons. There were 1145 upregulated genes that were both upregulated in the CAL27 miR-451a and CAL27_CTX groups. These genes were chosen as the upregulated genes for screening the genes activated by miR-451a. **(d)** PCA of the four samples showed a closer relationship between the genetic features of CAL27_CTX and CAL27 miR-451a than between the genetic features of the other two samples.

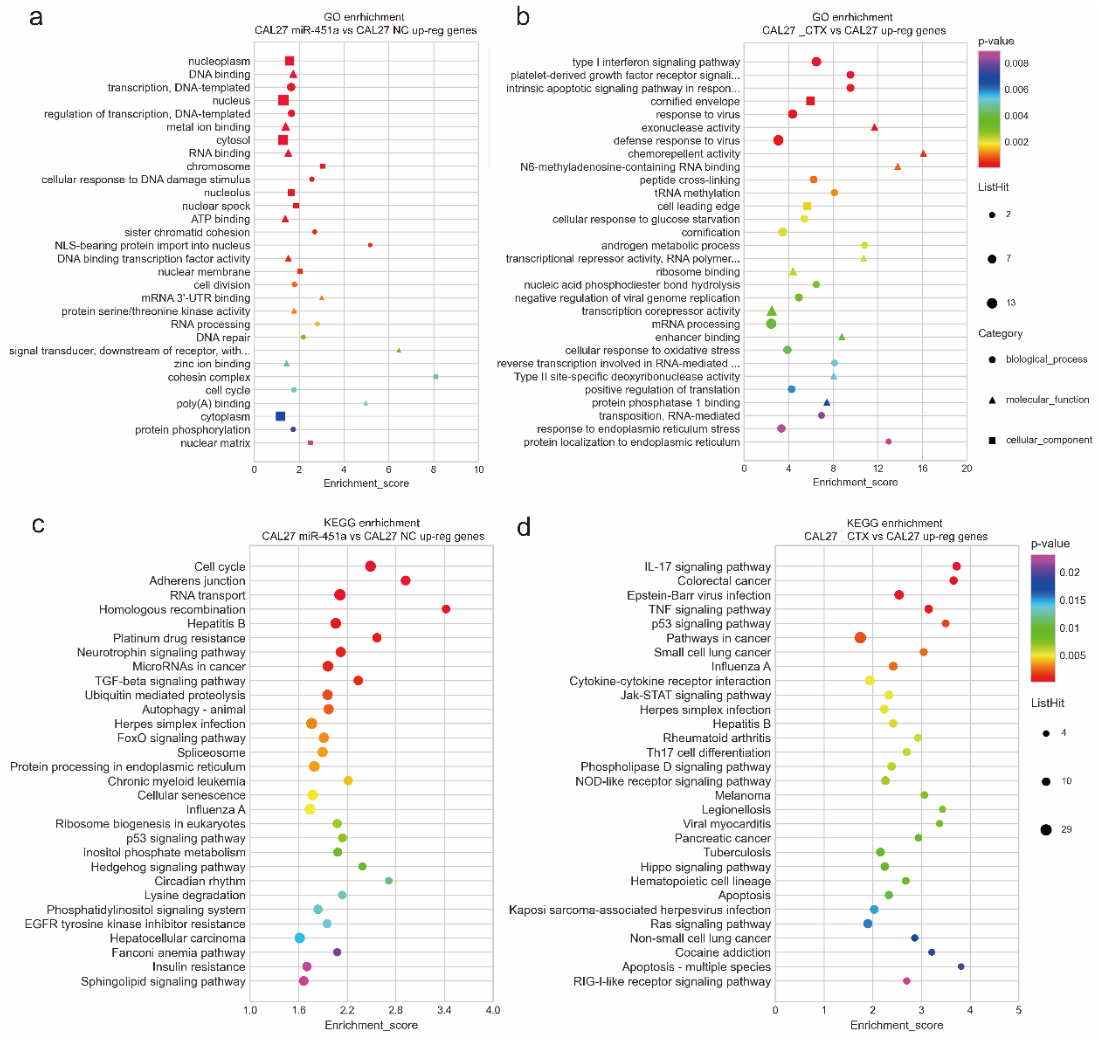

Figure S5. GO and KEGG analyses of the genes upregulated in the CAL27 miR-451a vs. CAL27 NC comparison and in the CAL27_CTX vs. CAL27 comparison. (a) GO analysis showing the genes upregulated in the CAL27 miR-451a group. **(b)** GO analysis showing the enrichment of genes upregulated in the CAL27_CTX strain. **(c)** KEGG analysis showing the enrichment of genes upregulated in the CAL27 miR-451a group. **(d)** KEGG analysis showing the genes whose expression was upregulated in the CAL27_CTX strain.

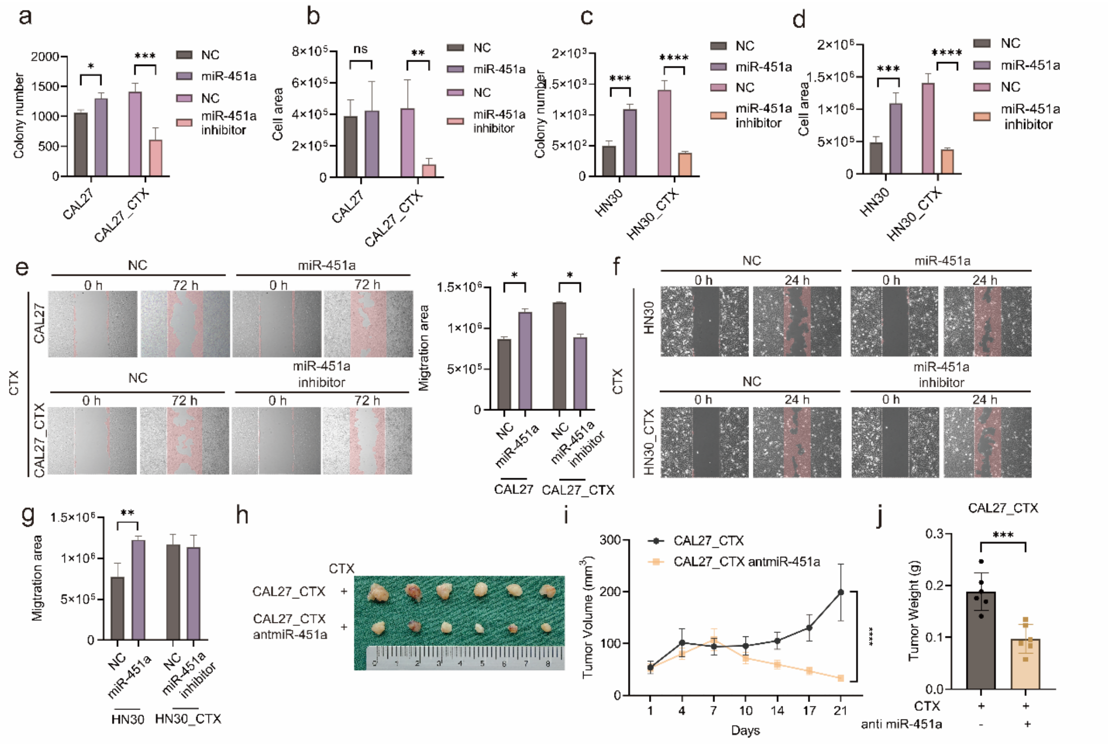

Figure S6. miR-451a enhanced HNSCC cell proliferation, migration and tumour growth upon treatment with cetuximab (a-d) Statistical analysis of the colony formation assay (a, c) and transwell (b, d) assay of miR-451a-overexpressing CAL27 cells and miR-451a-inhibited CAL27_CTX cells (a, b), miR-451a-overexpressing HN30 cells and miR-451a-inhibited HN30_CTX cells (c, d) treated with cetuximab. **(e-g)** Wound healing assay and statistical analysis of miR-451a-overexpressing CAL27 cells and miR-451a-inhibited CAL27_CTX cells (e), miR-451a-overexpressing HN30 cells and miR-451a-inhibited HN30_CTX cells (f, g) treated with cetuximab. **(h-i)** The tumour volume of the CAL27_CTX with antiagomiR-451a (CAL27_CTX antmiR-451a) group compared to that of the CAL27_CTX group (cetuximab treatment began at D7). **(j)** Tumour weights of the CAL27_CTX antmiR-451a group compared with those of the CAL27_CTX group. T tests were performed (a-e, g and j). Two-way ANOVA was performed to compare tumour growth at time points (i). ****$P < 0.0001$, ***$P < 0.001$, **$P < 0.01$, *$P < 0.05$; “ns” indicates no significance.

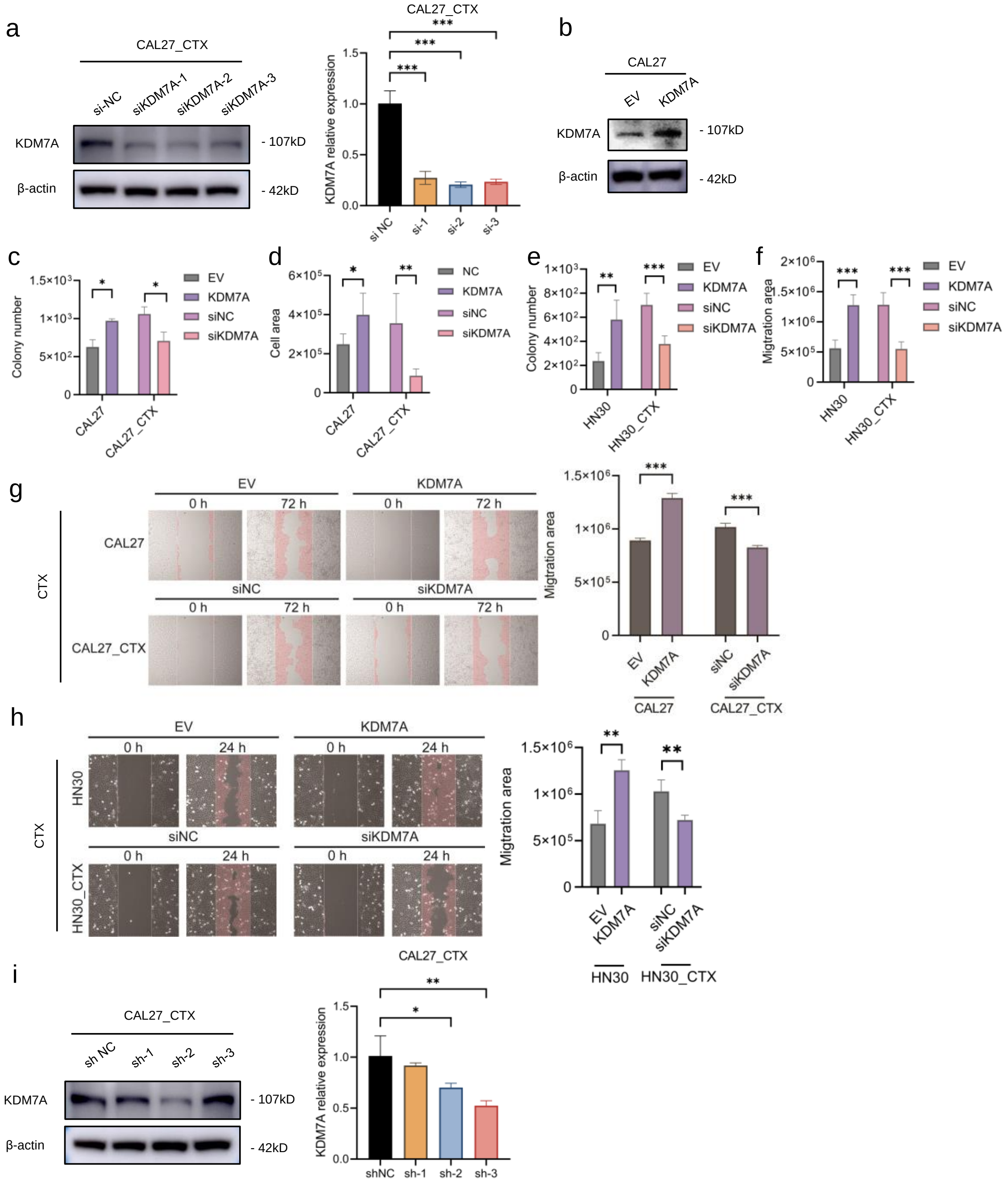

Figure S7. KDM7A enhanced HNSCC cells proliferation, migration and tumour growth upon treatment with cetuximab. (a) KDM7A knockdown via transient siRNA transfection. The western blot and qRT–PCR were performed to detect KDM7A expression at the protein and mRNA level. si-2 was eventually chosen for use. **(b)** KDM7A overexpression by the CMV vector validated by western blot. **(c-d)** Statistical analysis of the colony formation assay and transwell assay results comparing KDM7A-overexpressing CAL27 cells with CAL27 EV and KDM7A-silenced CAL27_CTX cells with CAL27_CTX siNC treated with cetuximab. **(d)** Statistical analysis of the Transwell assay results comparing KDM7A-overexpressing CAL27 cells and KDM7A-silenced CAL27_CTX cells treated with cetuximab. **(e)** Statistical analysis of the colony formation assay results comparing KDM7A-overexpressing HN30 cells and KDM7A-silenced HN30_CTX cells treated with cetuximab. **(f)** Statistical analysis of the Transwell assay results comparing KDM7A-overexpressing HN30 cells and

KDM7A-silenced HN30_CTX cells treated with cetuximab. **(g-h)** Wound healing assay was performed to compare KDM7A-overexpressing CAL27 cells and KDM7A-silenced CAL27_CTX cells (g), KDM7A-overexpressing HN30 cells and KDM7A-silenced HN30_CTX cells (h) treated with cetuximab. **(i)** KDM7A was knocked down via lentivirus transduction using shRNA. Western blot and qRT–PCR analyses were performed to detect KDM7A expression at the protein and mRNA level. sh-2 was eventually chosen for in vivo experiments. One-way ANOVA was performed (a and i). *T* tests were performed (c-h). ****$P < 0.0001$, ***$P < 0.001$, **$P < 0.01$, *$P < 0.05$; “ns” indicates no significance.

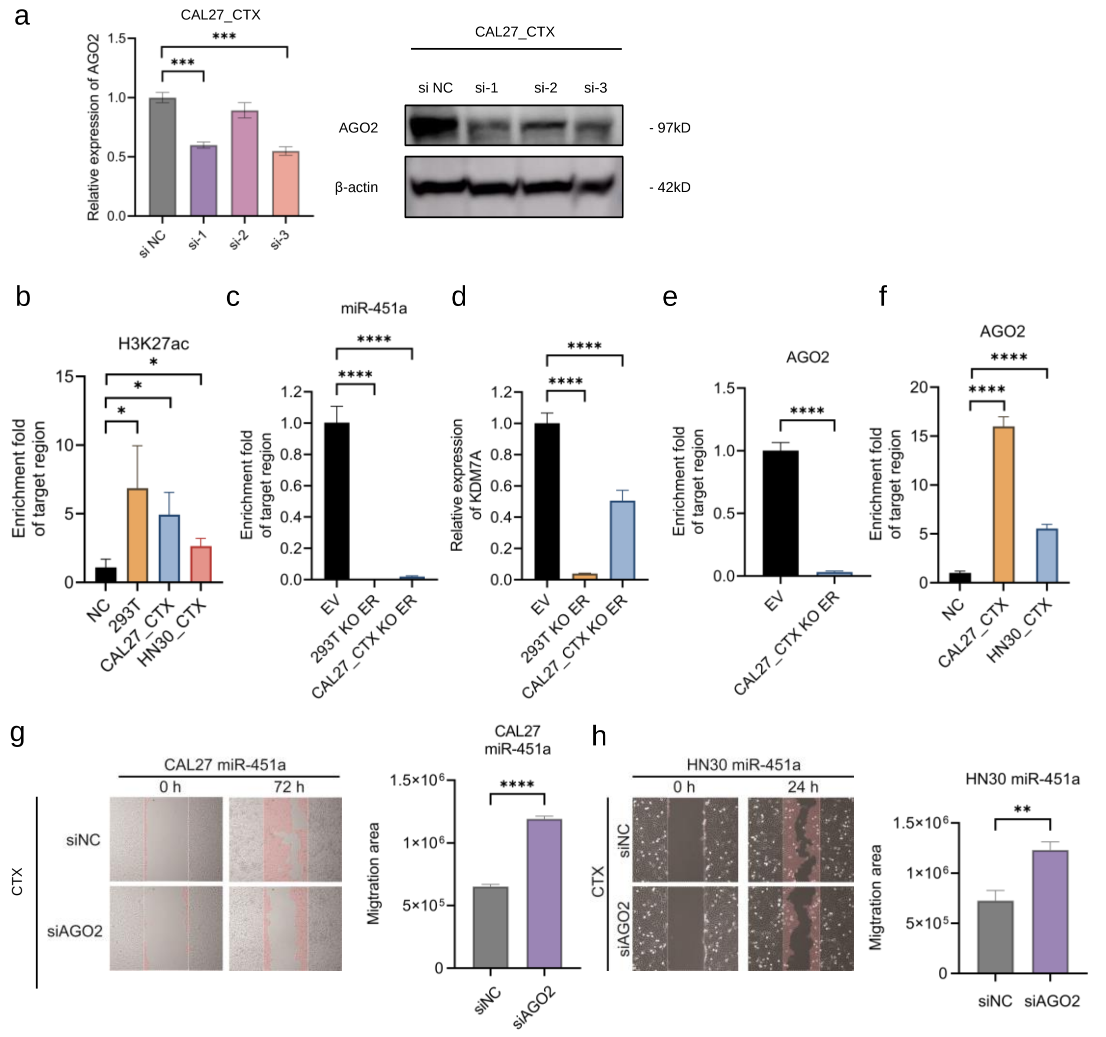

Figure S8. miR-451a enhanced KDM7A expression in an AGO2-dependent manner. (a) AGO2 was knocked down via transient siRNA transfection. The western blot and qRT–PCR were performed to detect AGO2 expression at the protein and mRNA level. si-3 was used to knock down AGO2. **(b)** ChIP–qPCR was performed using an H3K27ac antibody. **(c)** ChIRP-qPCR was performed using a miR-451a probe. The fold enrichment showed the interaction level between miR-451a and its target region. **(d)** KDM7A levels detected by q-PCR in enhancer-knockout 293T and CAL27_CTX cells. **(e-f)** ChIP–qPCR assay using an anti-AGO2 antibody to detect fold enrichment of the target region in CAL27_CTX KO ER cells (e) and in CAL27_CTX and HN30_CTX cells (f). **(g-h)** Wound healing assays in CAL27 miR-451a (g) and HN30 miR-451a (h) cells with and without siAGO2 knockdown. One-way ANOVA was performed (a). T tests were performed (b-h). ****P < 0.0001, ***P < 0.001, **P < 0.01, *P < 0.05; "ns" indicates no significance.

Supplemental tables

Table S1. The primers used for qRT–PCR analysis.

Gene	Primer sequences
hsa-miR-223-3p	5’- TGTCAGTTTGTCAAATACCCCA -3’
hsa-miR-451a	5’- AAACCGTTACCATTACTGAGTT -3’
hsa-miR-20b-5p	5’- CAAAGTGCTCATAGTGCAGGTAG -3’
hsa-miR-363-3p	5’- AATTGCACGGTATCCATCTGTA -3’
hsa-miR-4732-3p	5’- GCCCTGACCTGTCCTGTTCTG -3’
hsa-miR-486-5p	5’- TCCTGTACTGAGCTGCCCCGAG -3’
ARID1B	forward 5’- GCAAGGTGTGAGTGGTTACTG -3'
	reverse 5’- GGACTGGGACGGCAGATACT -3’
ATXN1	forward 5’- CAGAACCAGTACGTCCACATTT -3'
	reverse 5’- GGCGTATTGCATGACGACCT -3’
CCNT2	forward 5’- CAGCGTCTCAATGTCTCTCAG -3'
	reverse 5’- GAGTCTGTTGAAGGTAAGCATCA -3’
DNAJC3	forward 5’- GGCTCGGTATTCCCCTTCCT -3'
	reverse 5’- AGTAGCCCTCCGATAATAAGCAA -3’
ENAH	forward 5’- TCTATCACCATACAGGCAACAAC -3'
	reverse 5’- GCACAGTTTATCACGACCTGA -3’
HECTD1	forward 5’- ATTGCTGGAATGGCTACAGATG -3'
	reverse 5’- AAGGGCTGGTAAGAAAGTGCG -3’
KDM7A	forward 5’- GTATCACTGTCCCAACTGTGC -3'
	reverse 5’- TTCTGTGTAGTCATGTCTGTGC -3’
PRPF39	forward 5’- ATGACTCTCCCAATGTGAATGC -3'
	reverse 5’- ACCCAGCCTGTAAAATCCTGA -3’
AGO1	forward 5’- GACATCCCTAAGATCGACGTGT -3'
	reverse 5’- CCACTTCCCGGTTGACTCTAC -3’
AGO2	forward 5’- TCCACCTAGACCCGACTTTGG -3'
	reverse 5’- GTGTTCCACGATTTCCCTGTT -3’
AGO3	forward 5’- ATTGATGAGCAACCAAGACCTC -3'
	reverse 5’- ACAAACACGGTATTTCCGTCTC -3’
AGO4	forward 5’- AACCAATTCGACTGTTAGCCAA -3'
	reverse 5’- TCTACGAGGCCGTTTTTCAGG -3’
U6	forward 5’- CTCGCTTCGGCAGCACATATACT -3'
	reverse 5’- ATTTGCGTGTCATCCTTGCGCA -3’
MALAT	forward 5’- CCTAACCAGGCATAACACAGAAT -3'
	reverse 5’- CGAATGGCTTTGTCTCCGAA -3’
NEAT1	forward 5’- GCATACGCAGCAGATCAGCAT -3'
	reverse 5’- CCCACAATATAGGCATTTACAAGG -3’
BIRC5	forward 5’- GCAATGTCTTAGGAAAGGAGATCA -3'
	reverse 5’- AGAGAAGCAGCCACTGTTACCA -3’
β-Actin	forward 5’- TCACCCACACTGTGCCCATCTACGA -3'
	reverse 5’- CAGCGGAACCGCTCATTGCCAATGG -3’

Gene	Primer sequences
KDM7A TARGET REGION	forward 5’- GCAGAGTGGTAACGAGCATAGGC -3'
	reverse 5’- AACTTGCCCAAGAGAACTGGTGAAG -3’

Table S2. The siRNAs and shRNAs used in this study.

No.	Sequences
si-KDM7A-1	AGCCAGCUGACACAAAGAUAUTT
si-KDM7A-2	UGGAUUUGAUGUCCCUAUUAUTT
si-KDM7A-3	GGAAUGUGGAUACCAUGUCAATT
si-AGO2-1	CGUCCGUGAAUUUGGAAUCAUTT
si-AGO2-2	ACAGAUUCCCAAAGGGUAAAGTT
si-AGO2-3	AGGAUCGCAUCUUCAAGGUTT
sh-KDM7A-1	AGCCAGCUGACACAAAGAUAUdTdT
sh-KDM7A-2	UGGAUUUGAUGUCCCUAUUAUdTdT
sh-KDM7A-3	GGAAUGUGGAUACCAUGUCAAdTdT

Table S3. Primary antibodies used in this study.

Antigen	Manufacturer	Species	Cat. No.	Application
KDM7A	Abcam	Rabbit	ab230894	1:1000 for WB<br>1:200 for IHC
AGO2	ABclonal	Rabbit	A19709	1:1,000 for WB, 1:100 for RNA pull-down and ChIP
Histone 3	Proteintech	Rabbit	17168-1-AP	1:1,000 for WB
H3K27ac	ABclonal	Rabbit	A7253	1:100 for ChIP
GAPDH	ABclonal	Mouse	AC002	1:1,000 for WB
β-Actin	ABclonal	Rabbit	AC026	1:100,000 for WB

Abbreviations: WB, immunoblotting; IHC, immunohistochemistry

Table S4. Relationships between the miR-451a expression level and clinicopathologic features (n=87)

Characteristics	No.	Percent (%)	miR-451a relative expression (Mean ± SD)	Statistic value	*P* value
Age (years)					
≥60	61	70.12	2.06 ±1.23	*t*=-1.264	0.21
<60	26	29.89	1.71 ±1.1		
Gender					
Male	51	58.62	2.2 ±1.25	*t*=-2.327	0.22
Female	36	41.38	1.61 ±1.05		
Smoking history					
Nonsmoker	37	42.53	2.05 ±1.25	*t*=-0.595	0.55
Smoker	50	57.47	1.89 ±1.17		
Alcohol history					
Nondrinker	38	43.68	2.46 ±1.23	*t*=-0.724	0.47
Drinker	49	56.32%	2.83 ±1.18		
Tumour size (cm)					
≥4	34	39.08%	2.25 ±1.13	*t*=-1.852	0.07
<4	53	60.92%	1.77 ±1.21		
Lymph node metastasis					
pN0	54	62.07%	1.88 ±1.21		
pN1	20	22.99%	2.2 ±1.31	*F*=-0.541	0.58
pN2	13	14.94%	1.91±1		
TNM stage					
I	22	25.29%	2.16 ±1.31		
II	22	25.29%	1.81 ±1.22	*F*=0.354	0.79
III	24	27.59%	1.98 ±1.31		
IV	19	21.84%	1.86 ±0.93		
Pathological differentiation					
Well	42	48.28%	2.04 ±1.34		
Moderately	21	21.14%	1.77 ±1.08	*F*=-0.38	0.68
Poorly	24	27.59%	1.78±1.04		
Efficacy of cetuximab					
Nonresistant	47	54.02%	1.21 ±0.8	*t*=-8.509	0.0001
resistant	40	45.98%	2.83 ±0.97		
Recurrence					
Yes	17	19.54%	1.97 ±1.26	*t*=-0.031	0.98
No	70	80.46%	1.96 ±1.19		

Abbreviations: SD, standard deviation; pN, pathological lymph node metastasis; TNM stage, tumour-lymph node-metastasis stage.

Table S5. Relationships between the KDM7A expression level and clinicopathologic features (n=87)

Characteristics	No.	Percent (%)	KDM7A relative expression (Mean ± SD)	Statistic value	*P* value
Age (years)					
≥60	61	70.12	1.42 ±0.65	*t*=-1.557	0.12
<60	26	29.89	1.2 ±0.52		
Gender					
Male	51	58.62	1.46 ±0.62	*t*=-1.955	0.05
Female	36	41.38	1.2 ±0.59		
Smoking history					
Nonsmoker	37	42.53	1.46 ±0.63	*t*=-1.391	0.17
Smoker	50	57.47	1.27 ±0.6		
Alcohol history					
Nondrinker	38	43.68	1.43 ±0.56	*t*=-1.072	0.29
Drinker	49	56.32%	1.29 ±0.66		
Tumour size (cm)					
≥4	34	39.08%	1.35 ±0.61	*t*=-0.019	0.99
<4	53	60.92%	1.35 ±0.63		
Lymph node metastasis					
pN0	54	62.07%	1.36 ±0.69		
pN1	20	22.99%	1.37 ±0.51	*F*=-0.073	0.93
pN2	13	14.94%	1.29±0.44		
TNM stage					
I	22	25.29%	1.48 ±0.77		
II	22	25.29%	1.38 ±0.66	*F*=0.552	0.65
III	24	27.59%	1.25 ±0.5		
IV	19	21.84%	1.3 ±0.51		
Pathological differentiation					
Well	42	48.28%	1.71 ±0.52		
Moderately	21	21.14%	1.36 ±0.5	*F*=-1.332	0.27
Poorly	24	27.59%	1.44±0.71		
Efficacy of cetuximab					
Non-resistant	47	54.02%	1.14 ±0.52	*t*=-3.684	0.0001
resistant	40	45.98%	1.6 ±0.63		
Recurrence					
Yes	17	19.54%	1.42 ±0.51	*t*=-0.487	0.63
No	70	80.46%	1.34 ±0.64		

Abbreviations: SD, standard deviation; pN, pathological lymph node metastasis; TNM stage, tumour-

lymph node-metastasis stage.

Spreadsheet S1. Analysis of amplification exon sequencing of EGFR in CAL27 and HN30 cells and the corresponding cetuximab-resistant cell lines. See file amplification exon seq.xlsx

Spreadsheet S2. The intersection of downregulated genes from microarray comparisons of CAL27 miR-451a-overexpressing cells compared to CAL27 negative control cells and CAL27 cetuximab-resistant cells compared to CAL27 cells. See file: Intersection_down.xlsx

Spreadsheet S3. The intersection of upregulated genes from microarray comparisons of CAL27 miR-451a-overexpressing cells compared to CAL27 negative control cells and CAL27 cetuximab-resistant cells compared to CAL27 cells. See file: Intersection_up.xlsx

Spreadsheet S4. Analysis of Rank Ordering of Super-Enhancers on enriched common Peaks in CAL27 and HEK293T cells from ChIRP-seq results. See file ROSE_result.xlsx

Spreadsheet S5. miRANDA analysis of enriched common peaks in CAL27 and HEK293T cells from ChIRP-seq results. See file z_Result.csv
